# Impact of three commercial feed formulations on farmed gilthead sea bream (*Sparus aurata, L.*) metabolism as inferred from liver and blood serum proteomics

**DOI:** 10.1186/s12953-014-0044-3

**Published:** 2014-09-24

**Authors:** Stefania Ghisaura, Roberto Anedda, Daniela Pagnozzi, Grazia Biosa, Simona Spada, Elia Bonaglini, Roberto Cappuccinelli, Tonina Roggio, Sergio Uzzau, Maria Filippa Addis

**Affiliations:** Porto Conte Ricerche, S.P. 55 Porto Conte/Capo Caccia Km 8.400, Tramariglio, 07041 Alghero, Italy

**Keywords:** Gilthead sea bream, Aquaculture, Fish feed, Farmed fish, Liver proteins, Serum proteins, 2D DIGE, Mass spectrometry, Ingenuity pathway analysis, Proteomics

## Abstract

**Background:**

The zootechnical performance of three different commercial feeds and their impact on liver and serum proteins of gilthead sea bream (*Sparus aurata*, L.) were assessed in a 12 week feeding trial. The three feeds, named A, B, and C, were subjected to lipid and protein characterization by gas chromatography (GC) and liquid chromatography-tandem mass spectrometry (LC-MS/MS), respectively.

**Results:**

Feed B was higher in fish-derived lipids and proteins, while feeds C and A were higher in vegetable components, although the largest proportion of feed C proteins was represented by pig hemoglobin. According to biometric measurements, the feeds had significantly different impacts on fish growth, producing a higher average weight gain and a lower liver somatic index in feed B over feeds A and C, respectively. 2D DIGE/MS analysis of liver tissue and Ingenuity pathways analysis (IPA) highlighted differential changes in proteins involved in key metabolic pathways of liver, spanning carbohydrate, lipid, protein, and oxidative metabolism. In addition, serum proteomics revealed interesting changes in apolipoproteins, transferrin, warm temperature acclimation-related 65 kDa protein (Wap65), fibrinogen, F-type lectin, and alpha-1-antitrypsin.

**Conclusions:**

This study highlights the contribution of proteomics for understanding and improving the metabolic compatibility of feeds for marine aquaculture, and opens new perspectives for its monitoring with serological tests.

**Electronic supplementary material:**

The online version of this article (doi:10.1186/s12953-014-0044-3) contains supplementary material, which is available to authorized users.

## Background

Compatibility of feeds with fish metabolism is paramount for optimal zootechnical performance of the aquaculture plant [1]. However, producing the optimal diet for carnivorous marine species such as the gilthead sea bream (*Sparus aurata*, L.) would require the transformation of large amounts of wild fish in fish meal and fish oil [2]. The high costs and the unsustainable overexploitation of the wild fish stock have generated an increasing demand for developing feed formulations incorporating alternative raw materials, involving the replacement of fish meal with cheaper proteins of vegetable plant origin, including soybean, lupin seeds, peas, and sunflower [3]. However, although vegetable substitutes have produced encouraging results for species such as the rainbow trout (*Onchorynchus mykiss*) [4,5], these do often have limited compatibility with the metabolism of marine fish [6,7], due to the paucity or total lack of essential nutrients including specific aminoacids such as lysine and methionine, and fatty acids such as eicosapentanoic acid (EPA) and docosahexaenoic acid (DHA), that add to the poor protein digestibility [5,8]. Finding the key nutritional integrations is therefore becoming paramount to improve the metabolic compatibility of sustainable feeds, fish quality and its certification that, in turn, will attract consumer interest in higher level products. To this aim, sustainable and cheap protein sources, such as meat byproducts including blood meal, bone meal and, more recently, poultry or porcine waste matter, can integrate plant-based meals with some of the essential nutrients present only in fish meal and not in vegetable sources. Additional solutions to improve growth and protein utilization are represented by supplementing feed components with crystalline essential amino acids [5,9-11].

Insights into the response of fish metabolism to dietary substitutions can be provided by investigating how the proteome of pivotal tissues of the organism is affected by nutritional changes [12,13]. With this purpose, numerous research groups have applied proteomic analysis to farmed fish biofluids or tissues such as serum, liver, muscle, and other organs [12-21]. In particular, liver has gained the greatest attention for studying the influence of feed composition on fish metabolism. In fact, liver can be considered as the main metabolic reactor of the body: over 10,000 biochemical reactions are estimated to occur in this organ at any given time point, including carbohydrate, fat, and protein metabolism, storage of vitamins and minerals, and this organ is also known to possess a number of regulatory functions. Liver metabolism is considerably influenced by factors such as diet, environment, and stress, and might be affected by a wide range of xenobiotics and toxins [12]. In the past, Martin and coworkers [8,16] studied the changes occurring in the liver proteome as a consequence of different feeding regimens, including dietary plant protein substitution. Other authors investigated the liver proteome changes in gilthead sea breams exposed to low temperatures [22]; liver proteomics was also used to study the influence of handling and crowding as chronic stressors [18] and to study the effects of different contaminants, antibacterial, and antiparasitic agents [23,24].

Blood serum (or plasma) is a biological fluid of primary importance, being typically considered to be a ‘river’ of proteins and peptides bathing cells and tissues of the whole organism, acting as a mirror/reporter of physiological or pathological conditions [12,25]. Due to the many advantageous analytical traits, such as ease of sampling, handling and storage, elevated characterization, and limited processing required for analysis, serum is the preferred biological sample for monitoring a plethora of physiological and pathological parameters. In fish, serum proteomics has been successfully applied to investigate the response to numerous factors occurring in fish farming, including the response to domestication, the impact of various types of physical stressors, infections, and the administration of probiotics [12,17,19,21].

In this work, a twelve week feeding trial with three different commercial feeds was carried out on gilthead sea bream (*Sparus aurata*), the most relevant Mediterranean aquacultured fish species. Feeds were characterized for lipid composition by gas chromatography and for protein composition by shotgun proteomics followed by label-free quantitation. Liver tissue and blood serum of gilthead sea breams were subjected to proteomic characterization by 2D DIGE, nanoLC chip-cube ion trap tandem mass spectrometry, and pathway analysis. This study illustrates the contribution of proteomics in understanding the compatibility of feeds with fish metabolism, and discusses how the data gathered following these studies can provide inputs to improve feed formulations and, consequently, fish quality and economical value.

## Results

### Lipid and protein composition of the three feeds

Fatty acid (FA) composition of the three feeds is reported in Table 1. Feeds A and C were characterized by very high levels of linoleic acid (9c,12 t-18:2 n-6), and monounsaturated fatty acids, such as oleic (9c-18:1) and palmitoleic (9c-16:1) acids, probably as a consequence of high amounts of vegetable ingredients such as soy flour and oil. Feed B, on the other hand, showed a relatively high content of saturated FA (myristic 14:0, and palmitic 16:0) and essential long chain n-3 FA, such as eicosapentaenoic (EPA 20:5 n-3), docosapentaenoic (DPA 22:5 n-3) and docosaexaenoic (DHA 22:6 n-3), certainly derived from the higher amount of fish ingredients or fish oil components. Typical vegetable oils, such as oleic and linoleic acid, represented only a minor fraction of Feed B. It is worth noting that Feed A showed high contents of both DHA and EPA, which is in keeping with a substantial supplementation of fish oil to the vegetable matrix. On the other hand, Feed C, although based on raw materials similar to those of Feed A, contained the lowest percentages of DHA and EPA and the highest content of linoleic and oleic acids, likely due to a reduced supplementation with fish oil and a corresponding prevalence of vegetable lipids. Overall, these data fit with the raw materials listed in feed labels.

**Table 1:** **Fatty acid composition of the three feeds according to GC-MS analysis**

	**Common name**	**Feed A**		**Feed B**		**Feed C**	
**Fatty acid**		**%**	**SD**	**%**	**SD**	**%**	**SD**
14:0	myristic	5.45	0.01	8.33	0.00	2.55	0.02
16:0	palmitic	14.96	0.13	19.82	0.02	12.67	0.04
16:1 n-7	palmitoleic	5.56	0.03	8.63	0.02	3.29	0.02
18:0	stearic	3.52	0.01	3.94	0.01	2.87	0.01
18:1 n-9	n-9 oleic	16.67	0.05	12.01	0.01	17.72	0.11
18:1 n-11	n-11 oleic	2.31	0.01	3.41	0.00	1.79	0.04
18:2 n-6	linoleic	19.13	0.05	4.46	0.03	34.89	0.45
18:3 n-3 (linolenic acid)	α-linolenic	1.01	0.00	1.18	0.00	4.39	0.01
18:4 n-3	stearidonic	1.80	0.01	0.30	0.01	1.30	0.01
20:5 n-3	eicosapentaenoic	11.61	0.02	15.78	0.00	3.56	0.02
22:5 n-3	docosapentaenoic	1.44	0.01	2.05	0.04	0.36	0.00
22:6 n-3	docosahexaenoic	7.96	0.05	7.47	0.12	4.29	0.02

**Figure 1:**
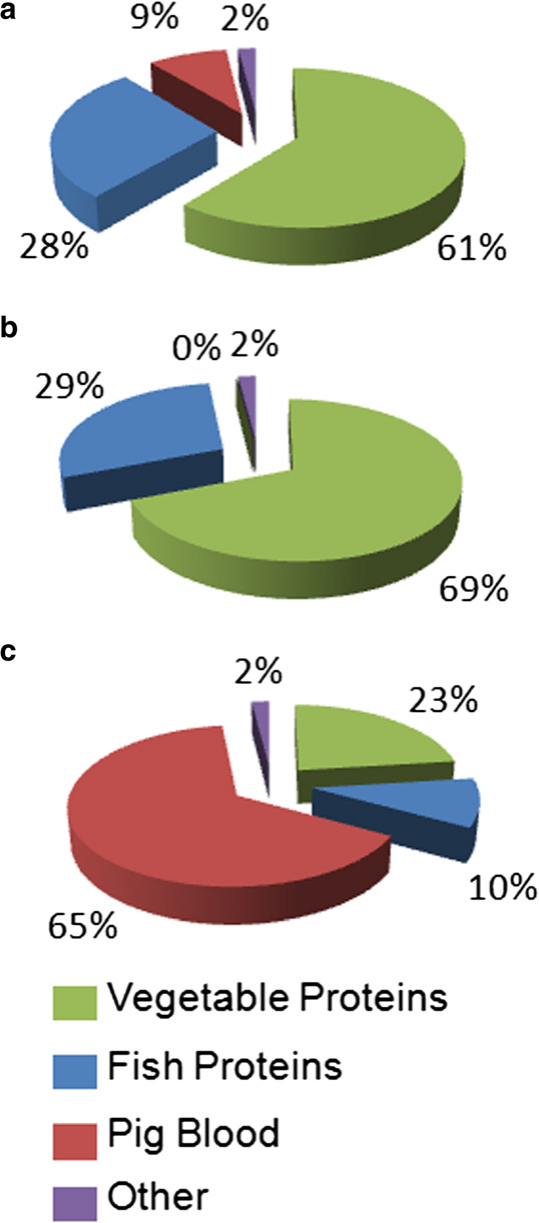
**Protein sources in feeds.** Pie charts illustrating the distribution of proteins according to their source in the three feeds used for this study and named **a**, **b**, and **c**. Protein sources are classified according to LC-MS/MS protein identification and ontology attribution.

With the aim of gathering information on their composition in terms of vegetable or animal proteins, feeds were also subjected to LC-MS/MS analysis. Protein composition percentages were assessed based on the protein identification ontology (Additional file 1). As illustrated in Figure 1, Feed B was the one with the highest relative fish protein composition (29%), followed by feed A (28%), and feed C (10%). Feed B, however, showed also the highest vegetable protein content (69% vs 61% and 23% for feeds A and C, respectively). Actually, Feed B did not contain any animal blood protein, which was by far the most relevant component of Feed C (65%), and accounted also for 9% of Feed A.

### Differences in fish growth performance according to different feeds

The impact of three different feeds (A, B, and C) on fish growth was initially assessed by evaluating the corresponding biometrical data (Table 2). A significantly higher average weight gain (AWG) (p < 0.05) was observed for Feed B (AWG = 121.19 ± 5,17 g) when compared to Feed C (AWG = 96,56 ± 7,21 g) and Feed A (AWG = 107,89 ± 7,71 g). Liver somatic indexes measured at the end of the trial accounted for the statistically significant differentiation of Feed B (LSI = 0,86 ± 0,11) from Feed A (LSI = 1,00 ± 0,17) and Feed C (LSI = 0,96 ± 0,11) (p < 0.05).

**Table 2:** **Biometrical results obtained on gilthead sea breams in the 12 week feeding trial**

	**Feed A**	**Feed B**	**Feed C**
IW (g)	268.54 ± 27.04	294.82 ± 28.05	276.61 ± 46.14
FW (g)	376.43 ± 45.09^a^	416.01 ± 47.65^b^	373.17 ± 55.28^a^
AWG (g)	107.89 ± 7.71^b^	121.19 ± 5.17^a^	96.56 ± 7.21^b^
LSI (%)	1.00 ± 0.17^a^	0.86 ± 0.11^b^	0.96 ± 0.11^a^

Values are reported as means ± S.E. (number of fish analyzed n = 45/feed for IW, FW, and AWG; n = 9/feed for LSI); a, b, and c indicate statistically different values (p < 0.05, Student’s t-test). IW: initial weight; FW: final weight; AWG: average weight gain; LSI: liver somatic index.

### Differential expression of gilthead sea bream liver proteins upon administration of the three feeds

To assess the impact of the three different feeds on the expression profile of sea bream liver proteins, a 2D DIGE analysis was implemented. The 2D DIGE approach was chosen in order to enable also detection of proteoforms differing in size and isoelectric point. In addition, the low level of sequencing and annotation of the *S. aurata* genome, and of fish species in general, was not ideal for carrying out a shotgun proteomics approach combined with label-free quantitation for differential protein profiling. 2D DIGE analysis of liver tissues was carried out by comparing expression levels at the beginning of the study (T0) and after 12 weeks of feeding with feeds A, B, and C, (T12A, T12B, and T12C, respectively). Unfortunately, the protein pattern of one of the Feed C gels presented technical problems and was not considered in the final analysis.

**Figure 2:**
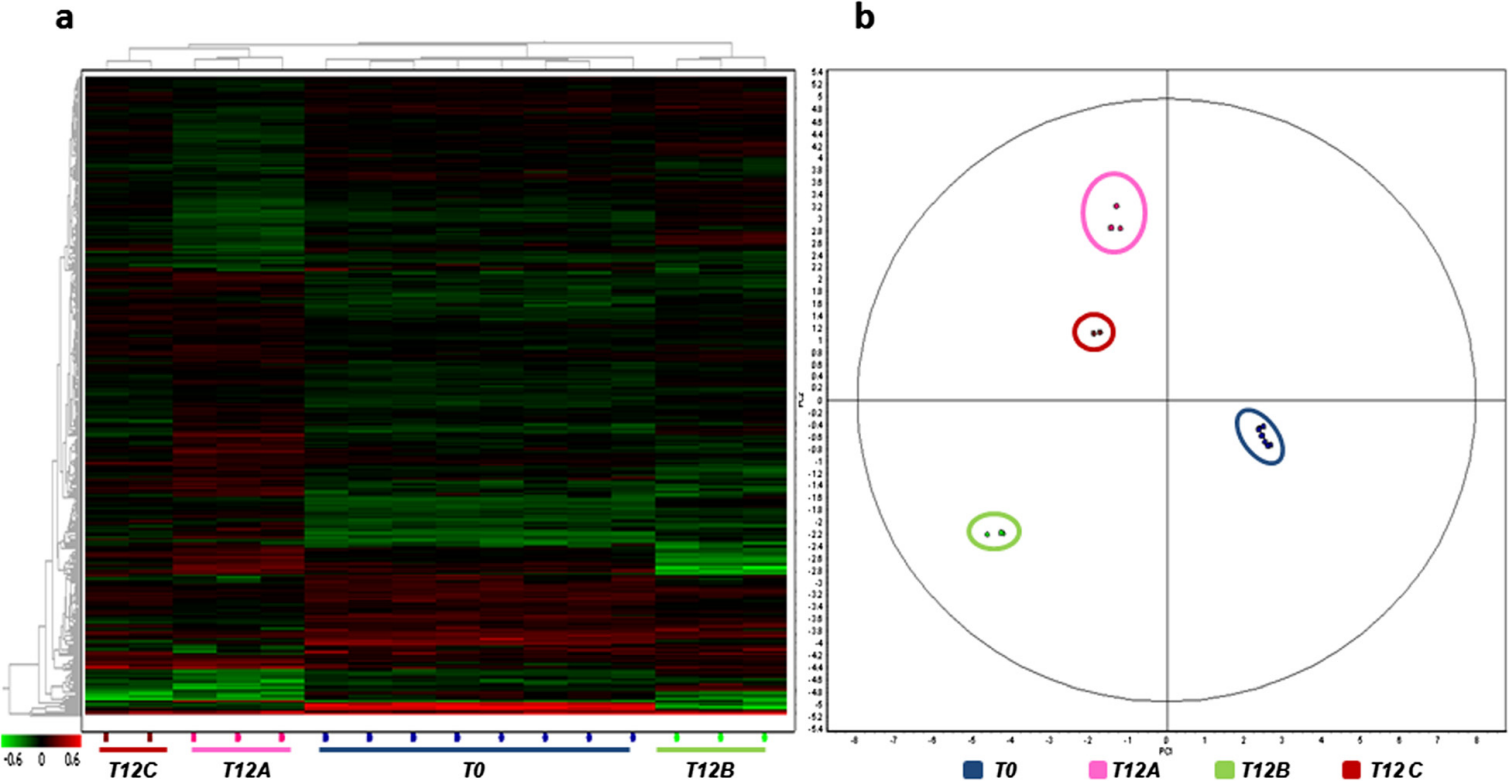
**Statistical analysis of 2D DIGE results.** Statistical analysis of 2D DIGE results. The figure illustrates the heat map **(a)** and the score plot **(b)** obtained upon comparison of the liver protein profiles of sea breams at T0 (blue) and after 12 weeks (T12) of feeding with feeds A (pink), B (green), and C (dark red). In the heat map **(a)**, each cell represents the differential protein expression trends, indicating increased expression in green and decreased expression in red. Clustering is performed according to the proteins (left dendrogram) and the sample (top dendrogram). In the score plot **(b)**, sample clustering according to the principal component analysis is reported.

As a result, 21, 24, and 11 statistically different spots (p < 0.05) with abundance levels above or below 1.5 fold were detected at T12A, T12B, and T12C, respectively. Multivariate analysis based on the principal component analysis (PCA) performed on all differential protein spots (p < 0.05) generated separate clusters (Figure 2b) differentiated by two principal components that distinguish the variance.

**Figure 3:**
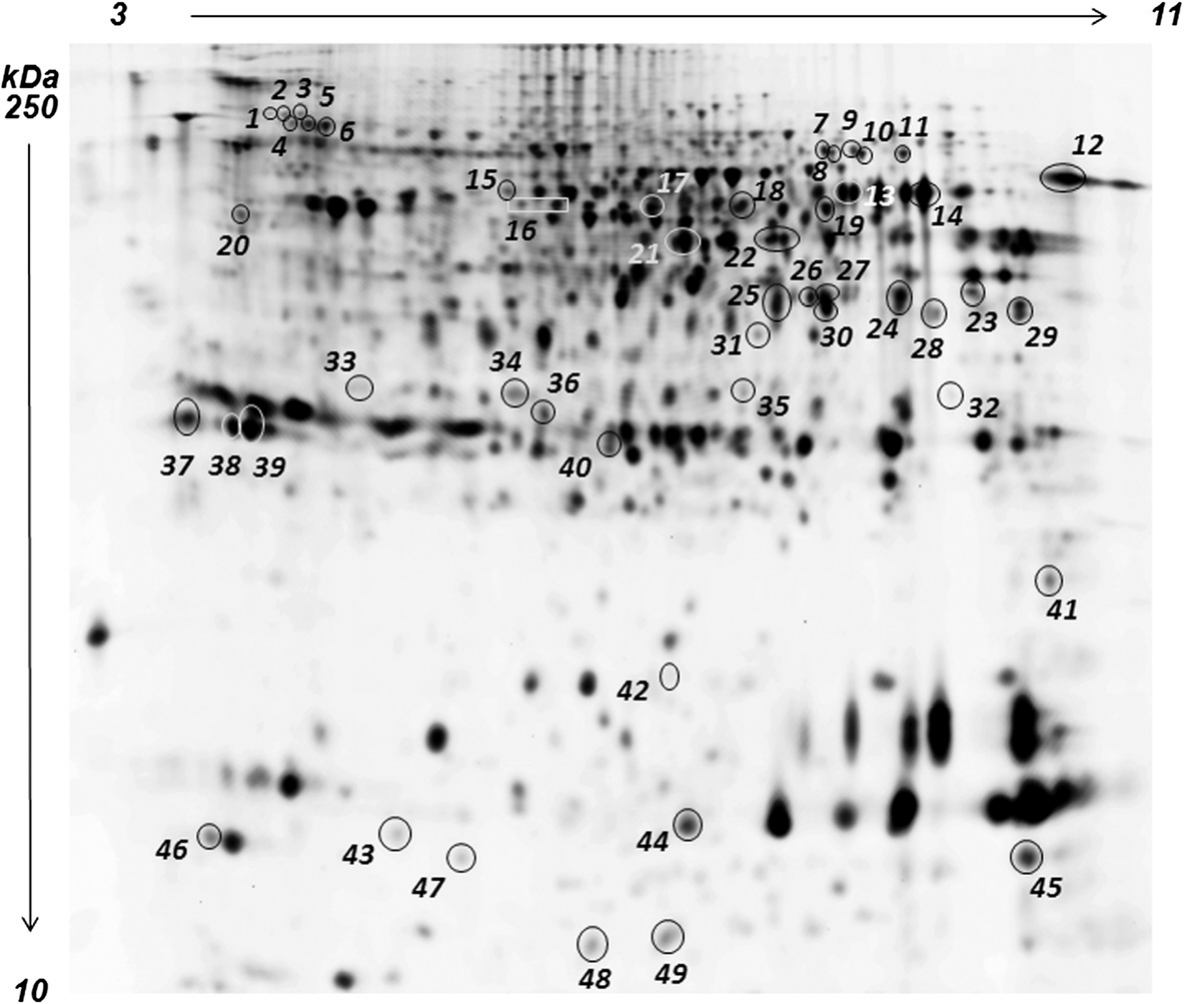
**Representative 2D PAGE of gilthead sea bream liver proteins.** Differentially expressed proteins are circled in the map, and information on their differential expression and identity is reported in Table 3.

As a first result, a significant divergence was seen for liver samples at T0 when compared to T12 for all feeds. In addition, among the three T12 groups, T12B clustered separately from T12A and T12C, in line with the biometrical results. Hierarchical clustering (HC) of differential spots and their levels of intensity (heat map) is presented in Figure 2a. The heat map and the hierarchical tree constructed using protein expression pattern similarities defined four separate groups (T12C, T12A, T0, T12B), highlighting also in this case a stronger separation of T12B from the other sample groups. Figure 3 reports a representative 2D PAGE of gilthead sea bream liver proteins indicating all the protein spots that underwent statistically significant differences in one of the sample groups, while Table 3 summarizes protein identifications and expression trends. Complete data on protein identifications are reported in Additional file 2. Spots that did not provide a valid protein identification are not reported.

**Table 3:** **Protein expression trends observed for T12A, T12B, and T12C vs T0, expressed as average ratios**

**Spot**	**T12A vs T0**	**T12B vs T0**	**T12C vs T0**	**Protein name**
1		1.7		Heat shock protein
7		1.52		UTP-glucose-1-phosphate uridylyltransferase
8		1.83		Alpha-amylase
9		1.77		Alpha-amylase
				UTP-glucose-1-phosphate uridylyltransferase
10		1.74		UTP-glucose-1-phosphate uridylyltransferase
12		1.55		Elongation factor 1-alpha
13			1.65	Betaine homocysteine methyltransferase
14			1.61	Betaine homocysteine methyltransferase
15	1.52	−4.58	2.01	Adenosylhomocysteinase
16	−2.02			Fumarylacetoacetate hydrolase
17	1.53			4-hydroxyphenylpyruvate dioxygenase
18		1.63	1.32	Isocitrate dehydrogenase
20	1.77			40S ribosomal protein SA
22	2			Malate dehydrogenase
23		−3.18		Fructose-bisphosphate aldolase
24	1.79	−1.91		Fructose-bisphosphate aldolase
27		−2.08		Fructose-bisphosphate aldolase
28		−1.83		Fructose-bisphosphate aldolase
29		−2.08		Malate dehydrogenase
				Betaine homocysteine methyltransferase
30	1.64			Fructose-bisphosphate aldolase
31	−1.59	−1.73		Guanine nucleotide-binding protein subunit beta-2 like 1
33		−2.06	−1.62	Prohibitin
				High choriolytic enzyme 1
34	−1.57			3-hydroxyanthranilate 34-dioxygenase
36	1.69			Betaine homocysteine methyltransferase
37			−1.08	Apolipoprotein A-IV
38	1.89			Apolipoprotein A-I
39	1.66			Apolipoprotein A-I
40	1.75			Betaine homocysteine methyltransferase
41		−2.1	1.41	Betaine homocysteine methyltransferase
42	−2.34	1.55		Peptidyl-prolyl cis-trans isomerase
43	1.9	−2.17		Fatty acid binding protein-like protein
44	1.7			Alpha-2 globin
45	−1.35	−4.35	−1.95	Alpha-1 globin
46	2.02	1.59		14 KDa Apolipoprotein
47		1.77		14 KDa Apolipoprotein
				Nucleoside disphosphate kinase
49	−2.2	−7.18	−1.87	Alpha-2 globin

Spots are numbered according to Figure 3. Details on protein identification by mass spectrometry are reported in Additional file 2. Non-significant changes and spots that did not provide a valid protein identification are not reported.

### Functional characterization of differential liver proteins: Ingenuity pathway analysis

All liver proteins showing statistically significant differences in expression from T0 to T12 for the three feeds, and their respective fold change values, were subjected to pathway analysis using the IPA software, with the aim of elucidating the main metabolic changes and representing them by networks. Since the IPA database builds on the literature generated on human and rodents, the UniProt codes for identified proteins were substituted with the UniProt codes of the closest human protein equivalents for the purpose of this analysis, as described previously for sea bass [26] and sheep [27]. The analysis was carried out considering the T12 for each feed and comparing its impact on the liver protein profile when compared to T0. For Feed A, the network scoring the best significance value was cell-to-cell signaling and interaction, inflammatory response, lipid metabolism (score 38). For Feed B, two networks produced a high score: cell-to-cell signaling and interaction, cellular function and maintenance, inflammatory response, with a score of 35, and cell death and survival, cellular compromise, cell cycle, with a score of 15. For Feed C, the highest scoring network (score 34) was lipid metabolism, molecular transport, small molecule biochemistry. Networks are reported in Additional file 3. Concerning top molecules, the highlighted differential proteins were 13 for Feed A (10 upregulated and 3 downregulated proteins), 18 for Feed B (10 upregulated and 8 downregulated proteins), and 12 for Feed C (9 upregulated and 3 downregulated proteins). Top molecules according to IPA are listed in Table 4.

**Table 4:** **Top scoring molecules according to IPA analysis obtained when comparing expression levels at T12 with expression levels at T0**

**Feed**	**Proteins**	**Exp.value**
A	Apolipoprotein A2 (APOA2)	2.020
	Aldehyde Dehydrogenase (ADH5)	2.000
	Malate dehydrogenase (MDH)	2.000
	Fatty Acid Binding Protein 1 (FABP1)	1.900
	Apolipoprotein A1 (APOA1)	1.890
	Aldolase B (ALDOB)	1.790
	Ribosomal 40S subunit (RPSA)	1.770
	Betaine-homocysteine S-methyltransferase 1 (BHMT)	1.750
	4-hydroxyphenylpyruvate dioxygenase (HPD)	1.530
	Adenosylhomocysteinase (AHCY)	1.520
	Fumarylacetoacetate hydrolase (FAH)	−2.020
	Guanine nucleotide-binding protein subunit beta-2-like 1 (GNB2L1)	−1.590
	Alpha-1 globin (HBA1)	−1.350
B	Apolipoprotein A2 (APOA2)	2.020
	Aldehyde Dehydrogenase (ADH5)	2.000
	Apolipoprotein A1 (APOA1)	1.890
	Alpha-amylase (AMY1A)	1.830
	Nucleoside-diphosphate kinase (NME4)	1.770
	Ribosomal 40S subunit (RPSA)	1.770
	UTP-glucose-1-phosphate uridylyltransferase (UGP2)	1.740
	Heat shock protein (HSPB1)	1.700
	Isocitrate dehydrogenase (IDH)	1.630
	Elongation factor 1-alpha (EEF1A1)	1.550
	Adenosylhomocysteinase (AHCY)	−4.850
	Alpha-1 globin (HBA1)	−4.350
	Aldolase B (ALDOB)	−3.180
	Fatty Acid Binding Protein 1 (FABP1)	−2.170
	Betaine-homocysteine S-methyltransferase 1 (BHMT)	−2.100
	Malate dehydrogenase (MDH1)	−2.080
	Prohibitin (PHB)	−2.060
	Guanine nucleotide-binding protein subunit beta-2-like 1 (GNB2L1)	−1.730
C	Apolipoprotein A2 (APOA2)	2.020
	Adenosylhomocysteinase (AHCY)	2.010
	Aldehyde Dehydrogenase (ADH5)	2.000
	Fatty Acid Binding Protein 1 (FABP1)	1.900
	Apolipoprotein A1 (APOA1)	1.890
	Aldolase B (ALDOB)	1.790
	Ribosomal 40S subunit (RPSA)	1.770
	Betaine-homocysteine S-methyltransferase 1 (BHMT)	1.650
	Isocitrate dehydrogenase (IDH)	1.320
	Alpha-1 globin (HBA1)	−1.950
	Apolipoprotein A4 (APOA4)	−1.800
	Prohibitin (PHB)	−1.620

### Differential expression of gilthead sea bream blood serum proteins upon administration of the three feeds

In order to assess the variation of serum protein levels following changes in feeding formulations, fish were sampled at T0 and at T12A, T12B, and T12C. Proteins from all samples were then compared for protein levels by 2D DIGE. As a result, 14, 13 and 8 differential spots were detected at T12A, T12B, and T12C, when compared to T0, respectively.

**Figure 4:**
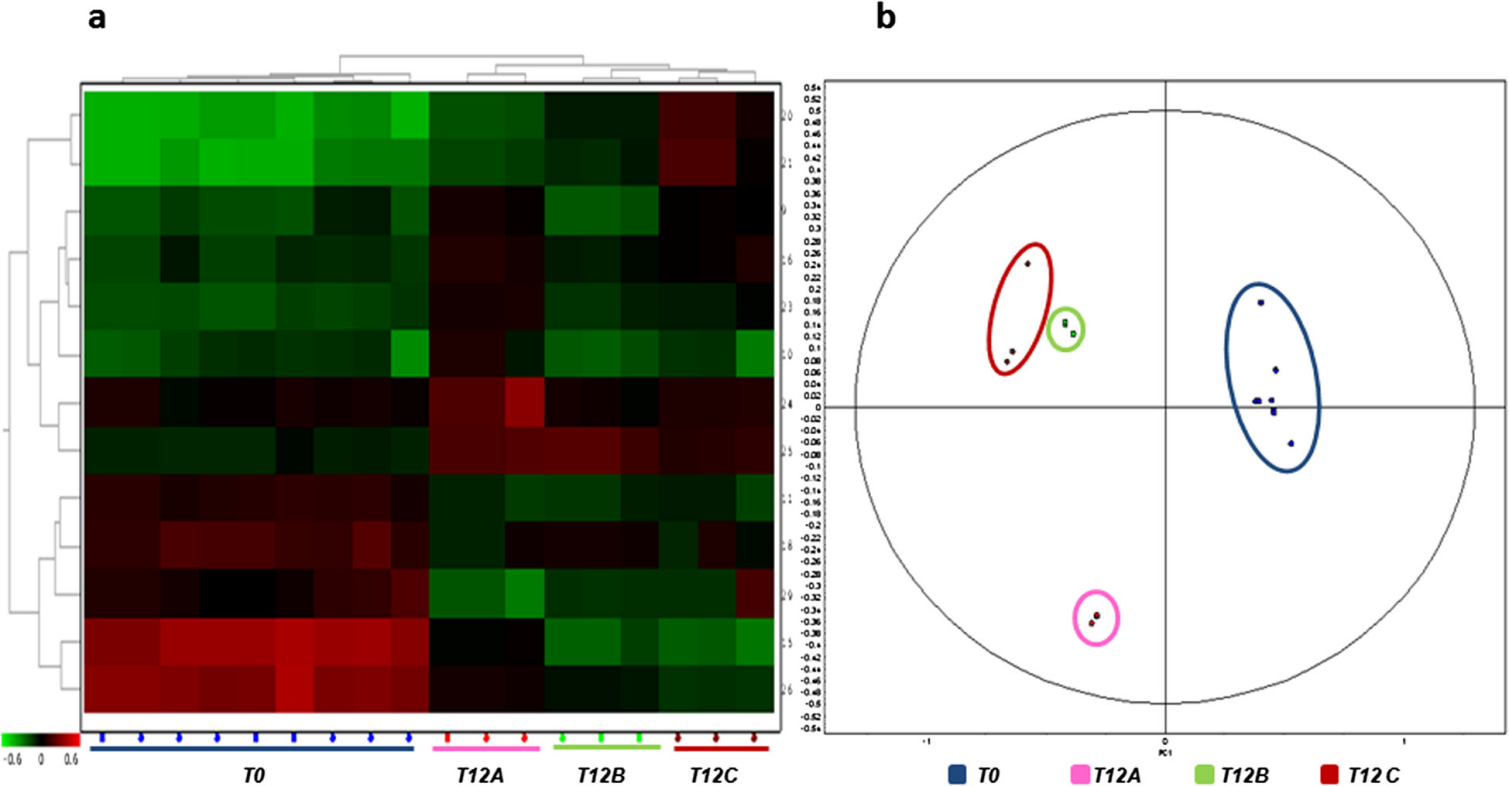
**Statistical analysis of 2D DIGE results obtained for fish serum.** The figure illustrates the heat map **(a)** and the score plot **(b)** obtained upon comparison of the serum protein profiles of sea breams at T0 (blue) and after 12 weeks (T12) of feeding with Feeds A (pink), B (green), and C (dark red). In the heat map **(a)**, each cell represents the differential protein expression trend, indicating increased expression in green and decreased expression in red. Clustering is performed according to the proteins (left dendrogram) and the sample (top dendrogram). In the score plot **(b)**, sample clustering according to the principal component analysis is reported.

**Figure 5:**
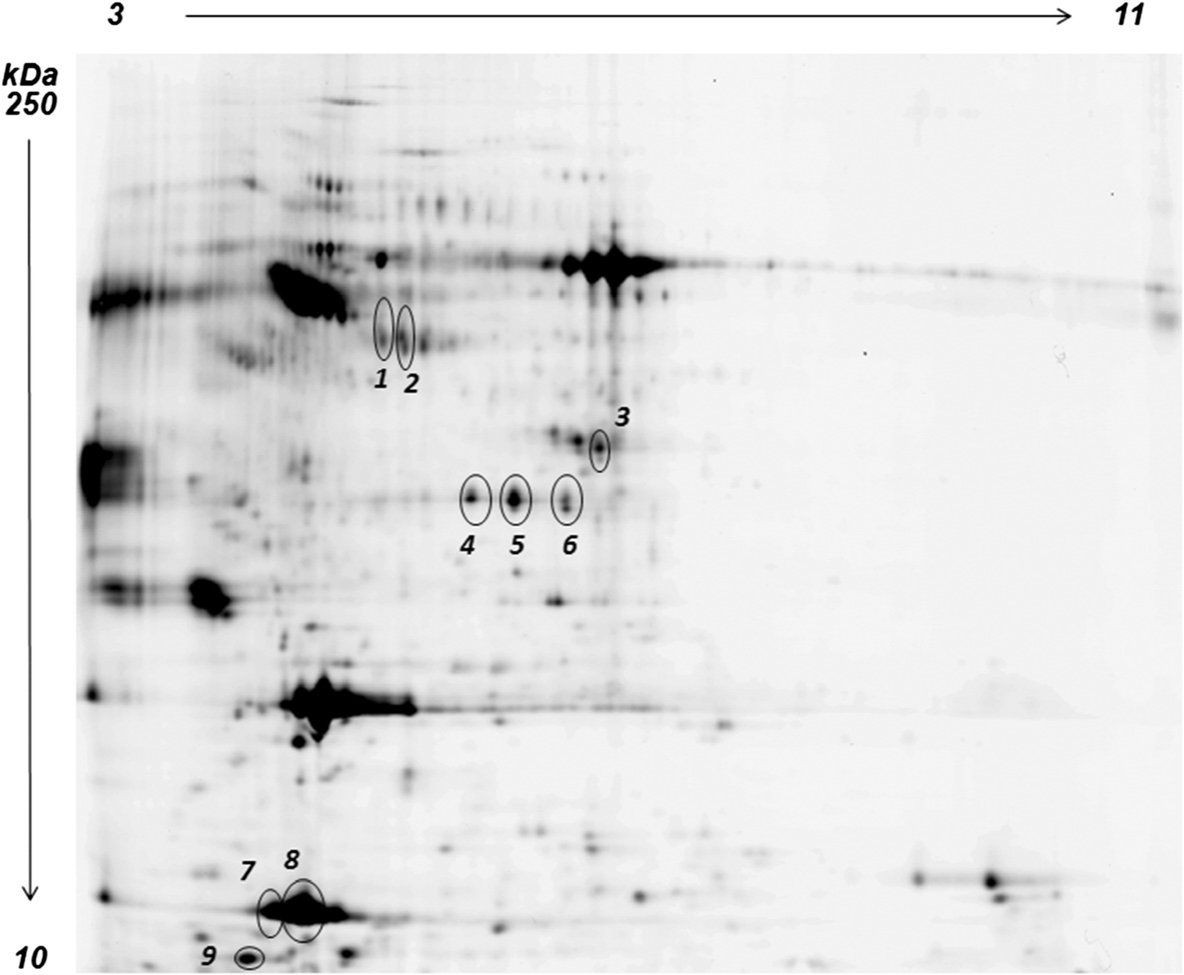
**Representative 2D PAGE of gilthead sea bream serum proteins in the 3 to 11 pH range.** Spots showing a differential abundance in T12A, T12B and T12C, and having a valid protein identification are circled in the map, and information on their changes and identities is reported in Table 5.

HC of differential spots and their levels of intensity (heat map) is presented in Figure 4a. The heat map and the hierarchical tree constructed using protein expression pattern similarities defined four separate groups (T12A, T0, T12B, T12C). Multivariate analysis based on the PCA performed on all differential protein spots (p < 0.05) generated four separate clusters (Figure 4b). However, statistically differential spots seen among the three feeds were mainly very faint, low molecular weight spots, that did not provide a valid identification or corresponded to higher molecular weight proteins. Table 5 reports only the identities of all major spots identified, as indicated in Figure 5.

**Table 5:** **Serum protein expression trends observed for T12A, T12B, and T12C vs T0**

**Spot**	**T12A vs T0**	**T12B vs T0**	**T12C vs T0**	**Protein name**
1	**2.3**			**Alpha 1 antitrypsin**
2	**1.7**			**Alpha 1 antitrypsin**
3		**1.5**		**Transferrin (fragments)**
4		**1.9**		**Fibrinogen beta chain**
5	**1.8**	**2.0**	1.3	**Fibrinogen beta chain**
6	1.4		**1.6**	**Fibrinogen beta chain**
7		**1.5**		**14 kDa apolipoprotein**
8	**1.5**	1.3	1.3	**14 kDa apolipoprotein**
9	**1.5**			**14 kDa apolipoprotein**

Spots are numbered according to Figure 5. Spots with average ratios +/- 1.5 are indicated in bold. Details on protein identification by mass spectrometry are reported in Additional file 4.

When examining the heat map and the PCA clustering in Figure 4, Feed C produced less significant variations at T12 when compared to Feeds A and B. On the other hand, Feeds A and B were clearly showing a higher impact on both biometric parameters and the liver proteomes. Therefore, a further experiment was carried out by using a narrower pH gradient, and with a higher number of replicates, to compare serum profiles at T12A and T12B, in order to highlight the differences existing between serum protein profiles in the two feeding trials at the end point. As a result, 20 statistically different spots (p < 0.05) with abundance levels above or below 1.5 fold were detected between T12A and T12B.

**Figure 6:**
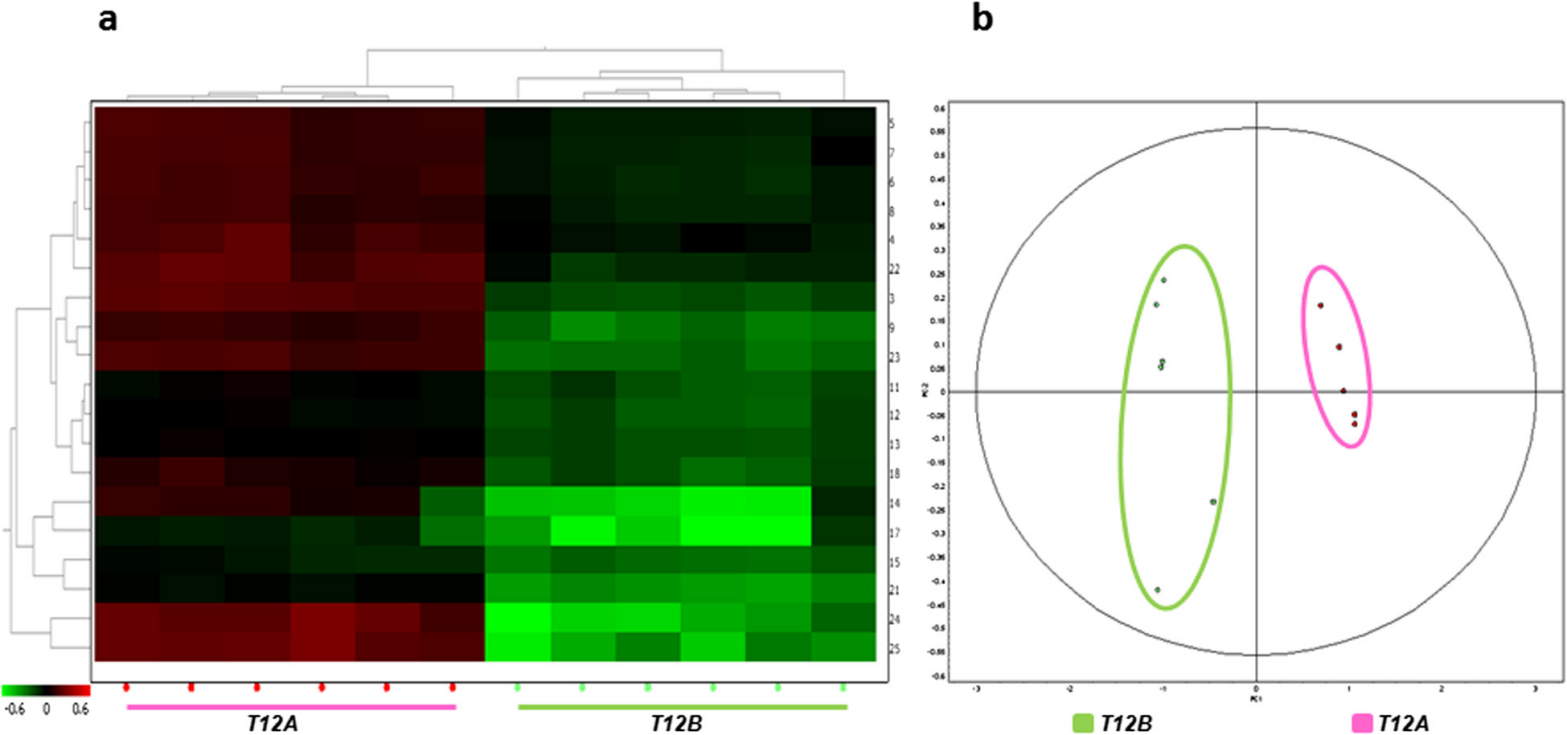
**Statistical analysis of 2D DIGE results.** The figure illustrates the heat map **(a)** and the score plot **(b)** obtained upon comparison of the serum protein profiles of sea breams at T12A (pink) and T12B (green). In the heat map **(a)**, each cell represents the differential protein expression trend, indicating increased expression in green and decreased expression in red. Clustering is performed according to the proteins (left dendrogram) and the sample (top dendrogram). In the score plot **(b)**, sample clustering according to the principal component analysis is reported.

**Figure 7:**
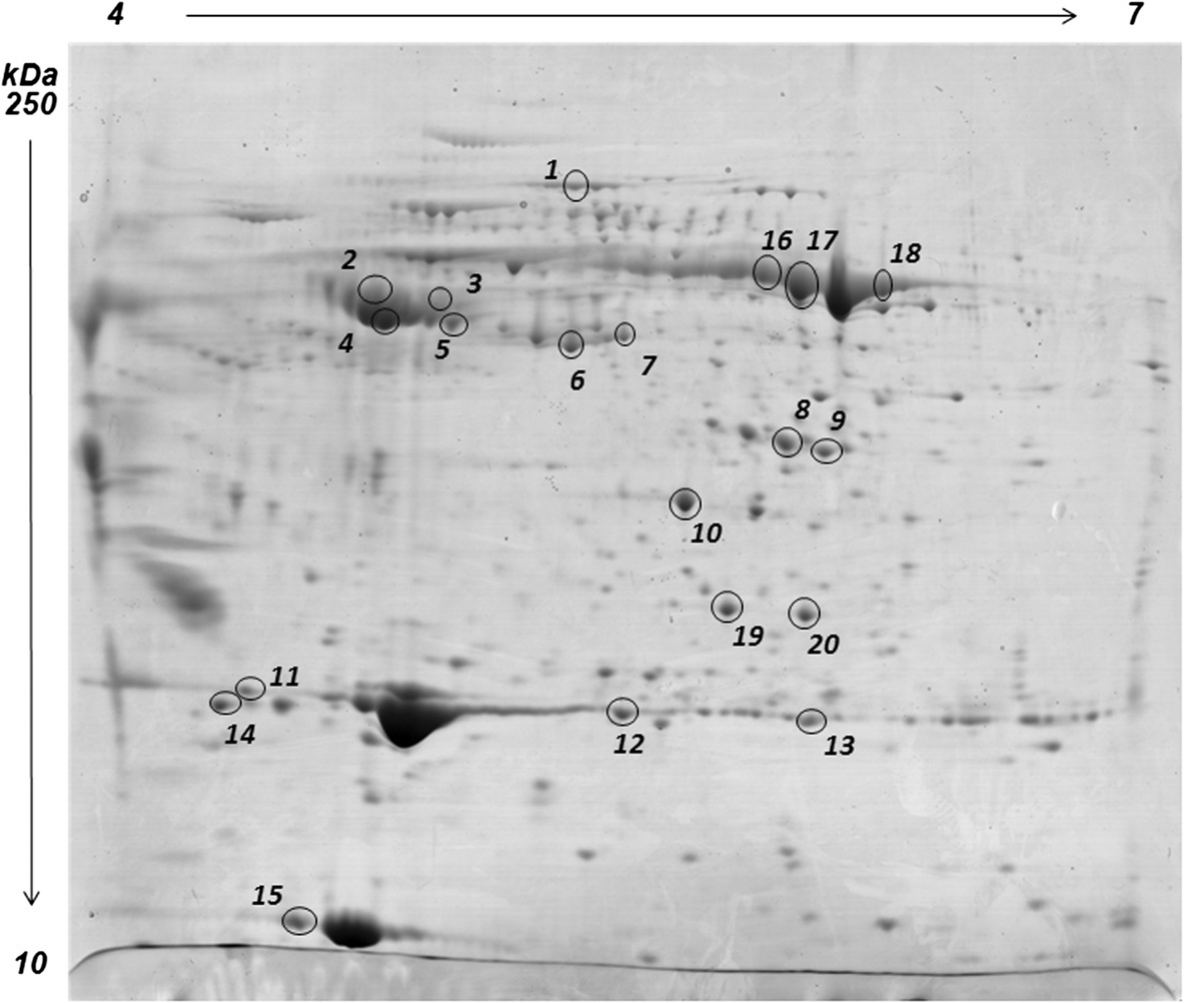
**Representative 2D PAGE of gilthead sea bream serum proteins in the 4 to 7 pH range.** Spots showing a differential abundance in T12A and T12B and a valid protein identification are circled in the map, and information on their changes and identity is reported in Table 6.

HC of differential spots and their levels of intensity (heat map) is represented in Figure 6a. The heat map and the hierarchical tree produced two clearly separated groups (T12A, T12B). The PCA comparing all samples generated the same clustering pattern, highlighting also in this case a strong separation of the two sample groups (Figure 6b). Statistically different protein spots were subjected to mass spectrometry identification. Figure 7 reports a representative map indicating all the major, statistically significant, differential spots, while Table 6 reports protein identities and their respective abundance changes. Detailed protein identifications are reported in Additional file 4.

**Table 6:** **Protein spots showing statistically significant differences in expression between T12A and T12B**

**Spot**	**Av. ratio T12A/T12B**	**Protein name**
1	1.59	Complement component c3
2	3.14	Alpha 1 antitrypsin
		Warm temperature acclimation-related 65 kDa protein
3	1.51	Warm temperature acclimation-related 65 kDa protein
4	2.96	Warm temperature acclimation-related 65 kDa protein
		Alpha 1 antitrypsin
5	1.5	Warm temperature acclimation-related 65 kDa protein
		Alpha 1 antitrypsin
6	1.87	Alpha 1 antitrypsin
7	3.86	Alpha 1 antitrypsin
8	−1.86	Transferrin (fragments)
9	−2.25	Transferrin (fragments)
10	−2.72	Fibrinogen beta chain
11	1.93	Apolipoprotein A-1
12	4.73	Apolipoprotein A-1
13	4.19	Apolipoprotein A-1
14	2.54	Apolipoprotein A-IV4
15	−3.3	14 kDa apolipoprotein
16	1.57	Transferrin
17	1.57	Transferrin
18	2.49	Transferrin
19	−1.77	F-type lectin 2
20	2.14	F-type lectin 2

Spots are numbered according to Figure 7. Details on protein identification by mass spectrometry are reported in Additional file 5.

Then, IPA was carried out for differential serum proteins, as previously done for liver proteins. However, in this case some significant differential proteins were either not assigned or provided controversial results, due to their unique functions in fish when compared to humans, mouse and rat (on which IPA is based), such as the warm temperature acclimation-related 65 kDa protein (Wap65). Consequently, the biological role of protein abundance differences was evaluated based on previous data available for fish in the scientific literature.

## Discussion

This study presents a comprehensive evaluation of the impact of three commercial feeds, designated as A, B, and C, on gilthead sea bream growth and metabolism, carried out by assessing protein abundance changes in liver tissue and blood serum at the end of a 12 week feeding trial (T0 vs T12A, T12B, and T12C, respectively). On a biometric scale, the three feeds produced differences in growth efficiency and in terms of liver somatic index, advantaging Feed B vs Feeds A and C (Table 2). In addition, the characterization of feeds revealed a higher amount of fish-derived lipids and proteins in Feed B when compared to Feeds A and C. The proteomic analysis of liver and serum of gilthead sea breams carried out at the end of the feeding trial highlighted a higher divergence of T12B from T12A and T12C, in agreement with the biometric observations. In addition, T12C fish diverged less from T0. Concerning this latter observation, it should be considered that all sea breams had been administered Feed C during the acclimation period preceding the trial, and therefore this group did probably undergo lesser metabolic changes when compared to T12A and T12B. As a further observation, T12A behavior was closer to T12C than to T12B, both in terms of growth and proteomic results. This is also consistent with the feed formulation, which was more similar for Feeds A and C in terms of lipid and protein composition.

### Liver proteomics

Similar changes in lipid metabolism were observed from T0 to T12 in all sea bream groups, although with few interesting differences induced by the three feed formulations investigated in this study. In liver, all three feeds induced a consistent and concerted overexpression of apolipoprotein A1 (APOA1) and 14 kDa apolipoprotein (apo-14), which is the fish homologue of apolipoprotein A2 (APOA2) [28]. This was also supported by the increase seen in serum apolipoprotein abundance. However, information on apolipoprotein functions are limited concerning lower vertebrates; in this respect, it should also be reminded that fish use lipids as a main source of energy, while higher vertebrates use carbohydrates [17,29]. A further consideration should be made concerning factors different than lipids that influence expression of apolipoproteins. In their study on the effect of dietary plant protein substitution in the rainbow trout, Martin and coworkers [5] commented on the role of phytoestrogens present in soy extracts on the increase in apolipoprotein levels. In our study, soy and soy oil were present in all feeds, and this may have accounted for slight differences in the extent of apolipoprotein overexpression. On the contrary, the three feeds were different in fatty acid composition as a consequence of high amounts of vegetable ingredients such as soy flour and oil in Feeds A and C (Table 1). Interestingly, fatty acid binding protein 1 (FABP1) showed a different behavior in Feed B when compared to Feeds A and C, being underexpressed in the former when compared to the latter. FABPs bind free fatty acids and their coenzyme-A derivatives, and are responsible for the transport and maintenance of fatty acids, being involved in their targeting to specific metabolic pathways, as well as in fatty acid signaling [30,31]. In keeping with the different FABP1 protein levels observed in liver of sea breams fed with Feed B as compared to those fed with Feeds A and C, the transcription factors that control FABP1 expression might be regulated according to the assortment of free fatty acid absorbed in the intestine. Isocitrate dehydrogenase (IDH) was also differently increased in feeds B and C, while no evidence of change was seen for Feed A. IDH is a key enzyme in lipid metabolism, being responsible for conversion of isocitrate to alpha-ketoglutarate and CO_2_ in the tricarboxylic acid cycle.

Concerning carbohydrate metabolism, among other observations, an increase in expression of alpha-amylase (AMY1A) and glycogen biosynthesis enzymes such as UTP-glucose-1-phosphate uridylyltransferase 2 (UGP2) was detected for Feed B, together with a decrease in glycolysis/gluconeogenesis enzymes, including fructose-bisphosphate aldolase (ALDOB) and malate dehydrogenase 1 (MDH1), when compared to feeds A and C, where an inverse behavior was seen. This may reflect the presence of higher amounts of amylaceous sources (starch) in this feed, due to presence of vegetable ingredients richer in this component, leading to a higher metabolic effort for its degradation and a consequent increase in glycogen storage, accompanied by an inhibition in glycolysis/gluconeogenesis pathways. Unfortunately, although the presence of starch sources is reported in the label for all three feeds, the relative proportion of all ingredients was not indicated.

Previous studies on plant-protein substitution in rainbow trout feeds have shown an increase in the activity of enzymes involved in amino acid metabolism [8,32]. Here, consequences on protein metabolism were also seen for gilthead sea breams, reflected by changes in the expression levels of betaine-homocysteine S-methyltransferase 1 (BHMT) and adenosylhomocysteinase (AHCY). These enzymes are involved in the superpathway of methionine degradation, as well as in the methionine salvage pathway. Specifically, BHMT is a cytosolic enzyme that catalyzes the conversion of betaine and homocysteine to dimethylglycine and methionine, respectively, while AHCY catalyzes the reversible hydrolysis of S-adenosylhomocysteine to adenosine and L-homocysteine. Also in this case, a different trend was observed for Feed B when compared to feeds A and C. Specifically, Feed B produced a decrease in expression of liver BHMT and AHCY, while feeds A and C produced an increase in the levels of both enzymes. A driver for the differential regulation of aminoacid metabolism enzymes may be represented by the significant differences in the animal protein sources present in the three feeds; in fact, Feed C and, in lesser amounts, Feed A, contain pig blood as a source of proteins, while in Feed B fish proteins are more abundant. In addition, both feeds contain vegetable protein sources. The unbalances in the optimal aminoacid ratio for the specific nutritional requirements of sea bream caused by the integration of fish protein sources are probably reflected on methionine metabolism, leading to the metabolic response we observed in the liver tissue. Therefore, the indications provided by the levels of these enzymes, mostly by BHMT, might be useful in pinpointing the correct integration of this essential aminoacid when designing the feed formulation, as well as for other aminoacid sources.

A further and equally important consideration concerning the changes in BHMT expression is related to the involvement of the methionine salvage pathway in the oxidative stress balance [22]. In fact, BHMT regulates the levels of S-adenosyl-methionine (SAM), which is crucial for methylation reactions, is a biosynthetic precursor of glutathione [33], and prevents homocysteine accumulation. The changes in BHMT levels can therefore be related also to glutathione biosynthesis, reflecting a different extent of oxidative stress caused on hepatocytes by the different feed formulations; more specifically, its decrease with Feed B when compared to its increase with Feed A and C indicates that a higher oxidative stress is exerted by the latter two feeds. Nevertheless, presence of an oxidative stress in all the feeds formulations tested is suggested by the significant increase in expression of aldehyde dehydrogenase (ADH), which is known to be associated with a protective action from oxidative stress [34]. In addition, it should be noted that the changes in expression levels seen for FABP and MDH1, although affecting other important metabolic pathways, are also related to glutathione oxidation/reduction pathways. Similar alterations in liver metabolism were observed by Ibarz and coworkers [22], who studied the response of the liver proteome in response to cold stress. Their studies highlighted alterations in protein expression that were linked to a reduced ability to respond to oxidative stresses under exposure to cold, and their identification of the metabolic pathways involved is consistent with our observations in this work.

Other proteins showing changes in expression levels were related to the nucleotide and small molecule metabolism, including nucleoside-diphosphate kinase (NME4), elongation factor-1-alpha (EEF1A1), and ribosomal 40S subunit (RPSA), all undergoing increases to different extents in the three feeds, and indicating a general increase in protein synthesis. Finally, a downregulation of prohibitin (PHB) was observed for feeds B and C. PHB has antiproliferative functions, and its decrease might be related to alterations in hepatocyte proliferation as a consequence of feeding, although it should be kept in mind that these are actively growing, young individuals, and this might as well be the result of physiological processes occurring as a consequence of body mass increase.

### Serum proteomics

In addition to liver, serum proteins were also evaluated in order to investigate on their possible differential abundance in this fluid as a consequence of feed composition. In fact, although serum collection is more problematic in fish when compared to other farmed animals, it may allow easier sampling and examination than internal organs or muscle. In addition, the identification of proteins showing a significant differential abundance under specific farming conditions may be useful for defining markers of fish wellness and/or other productive parameters of the farming plant. Similarly to liver, feeds A and C produced less divergent proteomic changes in serum, while Feed B led to a different result. In the first round of experiments comparing the serum protein profiles at T0 with those at the end of the feeding trial in all groups (T12A, T12B and T12C), only few major serum proteins showed changes in abundance, including alpha 1 antitrypsin (AAT), transferrin fragments, the fibrinogen beta chain, and apo-14.

The increase in apolipoproteins was consistent with the findings on liver tissue: a concerted increase of apo-14 was seen, higher in T12A. A concurrent increase in APOA1 was also seen, but only in T12A vs T12B in the case of serum. However, a decrease in a spot identified as apo-14 was seen in T12A vs T12B (spot 15, Figure 7), but in this latter case the larger spots of the isoelectric series were not affected; differences in abundance were seen only for minor spots, and further studies will be required to assess the possibility of post-translational modifications accounting for this phenomenon.

Wap65 was decreased at T12B when compared to T12A. Wap65 plays a key role in acclimation of fish to warm temperatures, having a possible role in maintaining proteins in their correct folding [35]. In addition, a role in response to pathogens, heavy metals, or other environmental stressors, has been reported [36,37]. The mammalian homologue of Wap65 is hemopexin, which is a serum transport protein with a role in transporting the haemoglobin prosthetic group, heme, to hepatocytes to facilitate its clearance [38,39]. In fish, it has been proposed that Wap65 is upregulated in order to scavenge heme with the aim of preventing bacterial growth [19,40]. The presence in feed A of pig haemoglobin can be accounted for these changes in Wap65 expression, especially when considering that sea breams were maintained at a constant water temperature during the whole feeding trial, and that abundance changes of Wap65 due to acclimation should have been minor.

Another protein undergoing variations in serum levels AAT. AAT is a secretory glycoprotein that in mammals functions as a serine proteinase inhibitor (serpin) [41], having multiple roles in inflammation and immune response [42]. Recently, its role in response to infections and inflammatory stimuli has been reported also in rainbow trout (*Oncorhynchus mykiss*), atlantic cod (*Gadus morhua*), and ayu (*Plecoglossus altivelis*) [43-45]. However, its implication in other types of stressors, such as oxidative stress deriving from a suboptimal diet, still need to be demonstrated. Fibrinogen beta chain was also consistently increased for all three feeds when compared to T0, and also in this case the correlation with liver stress due to unbalances in the feed composition can be hypothesized.

As a further interesting observation, abundance of full-length transferrin decreased in Feed B when compared to feed A. It is well known that transferrin is a major iron transporter in vertebrate blood; it absorbs iron in the gut and transports it between different body sites, acting as an iron shuttle and preventing a potentially toxic iron build up, although other functions are also known [40,46,47]. In our study, the increase seen in transferrin levels for feed A is likely related to an excess of iron deriving from the supplementation of the feed with pig hemoglobin, although other influences cannot be ruled out. Conversely, major transferrin fragments showed an inverse behavior when compared to the full-length protein, being increased in T12B. The release of transferrin fragments has been reported following lymphocyte reactions or mitogenic stimulations of goldfish kidney leukocytes [48]. These fragments, and not full-length transferrin, were able to induce the production of nitric oxide by LPS- stimulated goldfish macrophage cultures. This might suggest additional and different roles for transferrin fragments when compared to the full-length version of the protein. Alternatively, these may result from a higher turnover of full-length transferrin.

Another protein showing statistically significant variations was F-type lectin. Also in this case, changes in abundance were seen for spots having different isoelectric points; the more acidic one was higher in T12B, while the less acidic was higher in T12A. F-type lectins are fucose-binding proteins. Their biological role has not yet been clearly established, although evidence for an involvement in opsonization and immune response has been found in sea bass [49,50].

As a final consideration, however, it should be reminded that obtaining serum from fish still remains more problematic than for higher mammals, and this may favor degradation artifacts due to sample processing. Therefore, care should be taken in evaluating the implications of differential fragment abundance among the different conditions examined.

## Conclusions

Proteomics offers a valid approach to investigate the compatibility of feeds with the farmed fish metabolism. In this work, the MS/MS identification of differential spots in liver and serum maps provided useful insights into the influence of the different feed formulation on the lipid, carbohydrate, aminoacid and small molecule pathways, as well as on their impact on oxidative stress. In general, liver proteomics can help elucidate the pathways affected by feed substitutions and offers hints to improve quality, AWG and production yield. On the other hand, serum proteomics, although requiring further significant optimization and investigation efforts, may become a useful tool for the rapid monitoring of changes occurring in metabolism along farming, and offer opportunities for correction of the feeding regimen, both in the tested production lot as well as for future production lots. In addition, the information gathered can be used for valorization of high quality products, since fish is a source of essential fatty acids and is perceived as an healthy food by the consumers; therefore, adding value to the product can result into added value for the producer. In any case, however, the balance between feed price, weight gain, and product quality should always be kept in mind when considering the advantages in economical terms. To this aim, as supported by this work, proteomics can help the aquaculture industry to maintain a good relationship between production efficiency and product quality.

## Methods

### Experimental recirculating aquaculture system (RAS): the Blue Biotechnology Platform

The Blue Biotechnology platform at Porto Conte Ricerche is structured in three lines (seawater circuits), each composed of three 550 liter fiberglass tanks, independent from the others and controlled by dedicated mechanical and biological filtration systems. The feeding trial was carried out by dedicating each line to a test feed. Water flow was controlled automatically. Water temperature was set to 20 ± 0.5°C, pH to 7.8 ± 0.2, dissolved oxygen was fixed at 5.5 ± 1.0 mg/L, and salinity was measured to 37 ± 1‰. The system was kept partially sterilized with ozone and UV lamps (55 W, 1500–3000 L/hr).

### Fish, feeding regimens and husbandry conditions

Gilthead sea breams (*Sparus aurata*) were caught from a local fish farm (Alghero, Italy) and transferred to the RAS at Porto Conte Ricerche within 30 minutes. Fish with an average weight of 280 g were selected. Size variability at time zero was within 50 g overall. Sea breams were split in three lines (45 fish/line, 15 fish/tank) and acclimatized at 20°C. Then, all specimens were fed at 0.8% of biomass with 3 different commercial feeds, namely, Feed A, Feed B, and Feed C. Feed C was the same feed used during the acclimation period. The ration was calculated and readjusted according to changes in body weight. Each diet was distributed by hand once a day. The feeding trial lasted in total 85 days (about 12 weeks). At sampling dates (prior and at the end of the feeding trial), fish were anesthetized with 1,1,1-trichloro-2-methylpropan-2-ol (2% in marine H_2_O), and biometric data (body weight and length, liver weight) of each individual were taken. Initial weight (IW), final weight (FW), AWG and LSI were then calculated. The Student’s t-test was used for statistical analysis. Fish were then slaughtered in a mixture of ice and marine water. Blood samples were obtained by heart puncture.

### Characterization of commercial feeds

According to their label, commercial feeds had protein/fat relative ratios of 43/21, 45/24, and 43/21, for Feed A, Feed B and Feed C, respectively. All feeds integrated fish meal and oil with vegetables. The relative percentages of all feed ingredients were not specified by the producer; the feed composition label reported only the feed components in order of abundance, as follows. Feed A: fish powder, soy flour, fish oil, wheat flour, corn gluten, soy oil, magnesium sulfate; Feed B, certified “organic”: fish powder, green pea, fish oil, soy expeller, vitamins, minerals, antioxidants; Feed C: fish powder, soy flour, fish oil, hemoglobin, wheat flour, corn gluten, soy oil, magnesium sulfate. In our laboratories, specific tests were carried out to assess fatty acid composition and protein sources.

#### Lipids

Fatty acid composition analysis was performed on fatty acid methyl esters (FAME) of the total lipids extracted from powdered feed pellets according to the Folch method [51]. Briefly, approximately 10 mg of total lipid extract were methylated using KOH (2 N) in methanol. The samples were stirred for 1 min at ambient temperature, analyzed using an Agilent 7890A gas chromatograph (Agilent Technologes, Wilmington, DE) equipped with the flame ionization detector (FID), split/splitless injection port. A 100 m length and 0.25 mm internal diameter column was used (Supelco SP-2560). GC temperature program was set to 45°C (4 min), then increased to 175°C (13°C/min ramp, 27 min) and to 215°C (4°C/min ramp, 35 min). FAME standards were purchased from Nu-Check Prep (Elysian, MN, STD #463, #674).

#### Proteins

For characterization of protein composition, feed pellets were ground to powder and resuspended in 2% SDS in 20 mM Tris–HCl (pH 8.8) for 1 hour at room temperature, and then subjected to three cycles of freezing/thawing. After centrifugation in an Ultrafree MC Centrifugal Device (Millipore, now Merck Millipore, Billerica, MA, USA) proteins were quantified by using the BCA quantification kit (Thermo Fisher Scientific - Rockford, IL). SDS protein extracts were diluted to 200 uL with UA solution (8 M urea in 100 mM Tris–HCl, pH 8.8), loaded into the Microcon Ultracel YM-30 filtration devices (Millipore, now Merck Millipore, Billerica, MA, USA), and then processed according to the “FASP II” protocol [52] with minor modifications [53]. Briefly, samples were subjected to repetitive washings by filter centrifugations with buffers, reducing and alkylating agents, followed by overnight on-filter digestion with trypsin, final collection of peptides in acetonitrile (ACN) and formic acid, drying and reconstitution of the peptide mixture in 0.2% formic acid to a final concentration of 2 mg/mL.

LC–MS/MS analyses were performed on a Q-TOF hybrid mass spectrometer equipped with a nano lock Z-spray source and coupled on-line with a capillary chromatography system CapLC (Waters) as described before [54]. The peptide mixture was concentrated and washed onto a RP pre-column (Symmetry 300, C18, 5 mm, NanoEase, Waters) using 0.2% formic acid, and fractionated onto a C18 RP column (Nanoflow column 5 μm Biosphere C18, 75 μm × 200 mm, Nanoseparations) at a flow rate of 250 nL/min. The samples were fractionated using a linear gradient of eluent B (0.2% formic acid in 95% ACN) in eluent A (0.2% formic acid in 5% ACN) from 10 to 23% in 215 min and from 23% to 50% in 37 min. The mass spectrometer was set up in a data-dependent MS/MS mode where a full-scan spectrum was followed by tandem mass spectra, selecting peptide ions as the three most intense peaks of the previous scan. Argon was used as the collision gas, and collision energy depending on mass and charge of the precursor ion was applied. ProteinLynx software (Version 2.2.5), was used for analysis of raw MS and MS/MS spectra. Samples were analyzed in technical duplicate.

The peak lists for each sample duplicate were converted into a MGF file, which was analyzed by Proteome Discoverer (version 1.4; Thermo Scientific, Bremen, Germany) using an in-house Mascot server (version 2.3, Matrix Science) for protein identification according to the following criteria: Database UniProtKB/Swiss-Prot (release 2013_05), enzyme trypsin, taxonomy all entries, precursor mass tolerance 30 ppm, fragment mass tolerance 0.3 Da, methionine oxidation as dynamic modifications. The percolator algorithm was used for protein significance (p-value < 0.01) and for peptide validation (peptide confidence: q-value < 0.05), with only rank 1 peptides considered. Peptide and protein grouping according to the Proteome Discoverer’s algorithm were allowed, applying the strict maximum parsimony principle.

### Data analysis

Protein abundance was expressed by means of the normalized spectral abundance factor (NSAF). NSAF was calculated as follows: $\mathrm{NSAF}=\mathrm{SAFi}/{\displaystyle {\sum}_{i=1}^N SAFi}$, where subscript i denotes a protein identity and N is the total number of proteins, while SAF is a protein spectral abundance factor that is defined as the protein spectral counts divided by its length. In this approach, the spectral counts of each protein were divided by its length and normalized to the total sum of spectral counts/length in a given analysis [27,55].

### 2D DIGE analysis of liver and serum proteins

Fish liver was excised, weighed (for Liver Somatic Index determination, LSI), placed into a 10 mL screw-cap tube, and stored at −80°C until used. For protein extraction, a small portion of tissue was minced with a sterile scalpel, 100 mg were placed in 2 ml Eppendorf safe-lock tubes (Eppendorf, Hamburg, Germany), and immersed at 5% w/v in lysis buffer (7 M urea, 2 M Thiourea, 2% CHAPS, 0.5% IPG buffer pH 3–11 - GE Healthcare, Little Chalfont, UK). Each sample was processed with three cycles of 5 min at 30 oscillations/s in a TissueLyser mechanical homogenizer (Qiagen, Hilden, Germany) followed by freezing/thawing. All extracts were clarified for 15 min at 14,000 × rpm at 4°C, quantified with the 2D Quant kit (GE Healthcare), tested for quality and quantity by SDS-PAGE, and stored at −80°C until analysis. Blood was allowed to clot for two days at 4°C, since in many cases shorter times did not produce a complete clotting of the blood sample. The collected serum was then centrifuged for 10 min at 1,000 × rpm at 4°C, aliquoted and kept at −20°C until analysis. Serum samples were diluted 10 times in a lysis buffer containing 7 M urea, 2 M thiourea, 2% CHAPS, 0.5% IPG buffer pH 4–7 (GE Healthcare), quantified with the 2D Quant kit (GE Healthcare), tested for quality and quantity by SDS-PAGE, and stored at −80°C until analysis. For 2D DIGE, 6 sea breams for each condition (Feed A, Feed B, Feed C) were sampled, as well as 8 fish as initial controls. Sea bream liver samples were collected and pooled to minimize individual biological variability. The same procedure was followed for blood serum samples. We have previously demonstrated that the effect of inter-individual variability is less relevant than that imposed by different farming conditions in differentiating two or more fish groups [56,57]. However, the pooling approach was implemented in order to further minimize the effects of inter-individual variability due to the unavoidable minor differences among fish reared within the same group, while maximizing the overall response of each fish group to the specific dietary treatment. Sample labeling and 2D DIGE was carried out as described previously [58]. Fifty micrograms of protein from the test samples were labeled with the cyanine dye Cy3 or Cy5 (GE Healthcare), while a pooled internal standard sample was labeled with Cy2. The labeled proteins were mixed in suitable combinations. IPG buffer (GE Healthcare) and Destreak Rehydration Solution (GE Healthcare) were added to a final volume of 450 μl for each mix. First-dimension IsoElectric Focusing (IEF) was performed using 24-cm precast IPG strips in the pH ranges 3–11NL or 4–7 (GE Healthcare). The labeled sample mixtures were applied onto the strips by overnight passive rehydration at room temperature. The strips were focused on an IPGphor equipped with the Ettan™IPGphor3™ loading manifold (GE Healthcare) at 20°C for a total of about 90,000 Vh. After IEF, the strips were equilibrated, reduced, and alkylated by sequential incubation in 2% DTT and 2.5% iodoacetamide in 50 mM Tris–HCl (pH 8.8), 6 M urea, 20% glycerol, and 2% SDS, for 10 min each. The second dimension SDS-polyacrylamide gel electrophoresis was conducted on fixed 14% or 8-14% gradient polyacrylamide gels in a Ettan DALTtwelve electrophoresis system (GE Healthcare), following the manufacturer instructions.

### Image acquisition and statistical processing of data

After 2D electrophoresis, gels were scanned on a Typhoon Trio + image scanner (GE Healthcare) as described previously [58]. The scanned gel images were then transferred to the ImageQuant V5.2 software package (GE Healthcare), cropped, and exported to the DeCyder Batch Processor and differential in-gel analysis (DIA) modules (GE Healthcare) for statistical analysis. The results were compared and statistically evaluated by one-way analysis of variance (ANOVA) with the DeCyder biological variation analysis (BVA) module, applying the false discovery rate (FDR) to minimize the number of false-positive results. Protein spots with statistically significant variation (p < 0.05), showing a difference in volume of 1.5 fold, were selected as differentially expressed. Cluster analysis and visualizations were performed using the DeCyder extended data analysis (EDA) module. At the end of the analysis process, differentially expressed protein spots were selected for analysis by tandem mass spectrometry.

### Tandem mass spectrometry analysis

Preparative 2D PAGE gels were set up by loading 600 μg of protein extract into pH 3–11 NL (for liver), pH 4–7 (for serum), 24-cm IPG strips (GE Healthcare), which were then focused and subjected to 2-DE electrophoresis as described above. The gel was subjected to Coomassie R-250 staining [59], digitalized by scanning with an ImageScanner II (GE Healthcare), and matched to the 2D DIGE gel images generated for the three different feeds using the software Decyder 7.0, in order to track the spots to be excised for protein identification. Matched spots of interest were manually excised from the gels, destained, and subjected to overnight tryptic digestion as described previously [60]. Peptide mixtures were then collected by squeezing with ACN and centrifugation, then acidified, dried, resuspended in formic acid, and stored at −20°C.

LC-MS/MS analyses were performed on a XCT Ultra 6340 ion trap equipped with a 1200 HPLC system and a chip cube (Agilent Technologies, Palo Alto, CA), as described before [58]. Briefly, samples were concentrated and desalted on an enrichment column with formic acid, and peptides were fractionated on a C18 reverse-phase column directly coupled to a nanospray source. Data analysis software, provided by the manufacturers, was used to analyze MS/MS spectra and to generate a peak list which was analyzed by Proteome Discoverer (version 1.3, Thermo Scientific) using an in-house Mascot server (version 2.3, Matrix Science) for protein identification in the updated Trembl database, employing the Chordata (vertebrates and relatives) taxonomy and the following search parameters: precursor mass tolerance 300 ppm; fragment mass tolerance 0.6 Da; charge state +2, +3, and +4; enzyme trypsin; two missed cleavages; cysteine carbamidomethylation as static modification; and N-terminal glutamine conversion to pyroglutamic acid and methionine oxidation as dynamic modifications.

### Pathway analysis

Gene Ontology (GO) assignments and network analyses were carried out in the online software package IPA (version 9.0; Ingenuity Systems, Redwood City, CA). The list of protein identifications (IDs) with P values ≤0.05, together with their respective average ratio values, was imported into the online software package IPA and network analyses were performed with thresholds of 1.5 for RSC and 0.05 for P value. Fish UniProt IDs were replaced with the UniProt IDs for the closest human protein equivalents in order to enable the best exploitation of the knowledge-based IPA software, as described before for organisms not included in the IPA database [26,27]. To determine the biological processes, functions, pathways, and molecular networks most significantly altered during the three feeding trials, both over- and underrepresented proteins were defined as value parameters, all identifier types and data sources were selected in order to access all available information in the IPA database, and both direct and indirect relationships were considered.

## Additional files

Additional file 1:
**Excel file with shotgun proteomics data on feeds.**
Additional file 2:
**Detailed protein identifications of liver.**
Additional file 3:
**Protein networks generated by IPA software.**
Additional file 4:
**Detailed protein identifications of serum for the comparison of T12A, T12B and T12C vs T0.**
Additional file 5:
**Detailed protein identifications of serum for the comparison of T12A vs T12B.**

## Abbreviations

2D DIGE: Two-dimensional differential in-gel electrophoresis
2D PAGE: Two-dimensional electrophoresis
AAT: Alpha 1 antitrypsin
ACN: Acetonitrile
ADH: Aldehyde dehydrogenase
AHCY: Adenosylhomocysteinase
ALDOB: Fructose-bisphosphate aldolase
AMY1A: Alpha-amylase 1
ANOVA: One-way analysis of variance
apo-14: 14 kDa apolipoprotein
APOA1: Apolipoprotein A1
APOA2: Apolipoprotein A2
AWG: Average weight gain
BHMT: Betaine-homocysteine S-methyltransferase 1
BCA: Bicinchoninic acid
CHAPS: 3-[(3-Cholamidopropyl)-dimethylammonio]-1-propane sulfonate
DHA: Docosahexaenoic acid
DIA: Differential in-gel analysis
DPA: Docosapentaenoic acid
DTT: Dithiothreitol
EDA: Extended data analysis
EEF1A1: Elongation factor-1-alpha
EPA: Eicosapentanoic acid
FA: Fatty acid
FABP1: Fatty acid binding protein 1
FAME: Fatty acid methyl esters
FASP: Filter-aided sample preparation
FDR: False discovery rate
FID: Flame ionization detector
FW: Final weight
GC: Gas chromatography
GO: Gene ontology
HC: Hierarchical clustering
HPLC: High performance liquid chromatography
IEF: Isoelectric focusing
IPA: Ingenuity Pathway Analysis
IPG: Immobilized pH gradient
IDH: Isocitrate dehydrogenase
IW: Initial weight
LC: Liquid chromatography
LPS: Lipopolysaccharide
LSI: Liver somatic index
MDH1: Malate dehydrogenase 1
MS: Mass spectrometry
MS/MS: Tandem mass spectrometry
NME4: Nucleoside-diphosphate kinase
NSAF: Normalized spectral abundance factor
PCA: Principal component analysis
PHB: (Prohibitin)
Q-TOF: Quadrupole-time of flight
RAS: Recirculating aquaculture system
RP: Reverse phase
RPSA: Ribosomal 40S subunit
SAM: S-adenosyl-methionine
SDS: Sodium dodecyl sulphate
SDS-PAGE: Sodium dodecyl sulphate-polyacrylamide gel electrophoresis
T0: Sampling at time zero
T12A: Sampling at 12 weeks of feeding with feed A
T12B: Sampling at 12 weeks of feeding with feed B
T12C: Sampling at 12 weeks of feeding with feed C
UGP2: UTP-glucose-1-phosphate uridylyltransferase 2
Wap65: Warm temperature acclimation-related 65 kDa protein

## References

[1] FAO (2010). The state of world fisheries and aquaculture.

[2] Naylor RL, Goldburg RJ, Primavera JH, Kautsky N, Beveridge MC, Clay J, Folke C, Lubchenco J, Mooney H, Troell M (2000). Effect of aquaculture on world fish supplies. Nature.

[3] Carter CG, Hauler RC (2000). Fish meal replacement by plant meals in extruded feeds for Atlantic salmon, *Salmo salar* L. Aquaculture.

[4] Kaushik SJ, Cravedi JP, Lalles JP, Sumpter J, Fauconneau B, Laroche M (1995). Partial or total replacement of fish meal by soybean protein on growth, protein utilization, potential estrogenic or antigenic effects, cholesterolemia and flesh quality in rainbow trout, *Oncorhynchus mykiss*. Aquaculture.

[5] Vilhelmsson OT, Martin S a M, Médale F, Kaushik SJ, Houlihan DF (2004). Dietary plant-protein substitution affects hepatic metabolism in rainbow trout (*Oncorhynchus mykiss*). Br J Nutr.

[6] Watanabe T, Aoki H, Watanabe K, Maita M, Yamagata Y, Satoh S (2001). Quality evaluation of different types of non-fish meal diets for yellowtail. Fish Sci.

[7] Pereira TG, Oliva-Teles A (2003). Evaluation of corn gluten meal as a protein source in diets for gilthead sea bream (*Sparus aurata* L.) juveniles. Aquac Res.

[8] Martin SAM, Vilhelmsson O, Médale F, Watt P, Kaushik S, Houlihan DF (2003). Proteomic sensitivity to dietary manipulations in rainbow trout. Biochim Biophys Acta - Proteins Proteomics.

[9] Yamamoto T, Shima T, Furuita H, Suzuki N (2002). Influence of feeding diets with and without fish meal by hand and by self-feeders on feed intake, growth and nutrient utilization of juvenile rainbow trout (*Oncorhynchus mykiss*). Aquaculture.

[10] Martìnez-Llorens S, Vidal AT, Garcia IJ, Torres MP, Cerdà MJ (2009). Optimum dietary soybean meal level for maximizing growth and nutrient utilization of on-growing gilthead sea bream (*Sparus aurata*). Aquac Nutr.

[11] Kaushik SJ, Seiliez I (2010). Protein and amino acid nutrition and metabolism in fish: current knowledge and future needs. Aquac Res.

[12] Addis MF, Toldrá F, Nollet LML (2013). Proteomics in Foods. Proteomics Foods Princ Appl.

[13] Rodrigues PM, Silva TS, Dias J, Jessen F (2012). Proteomics in aquaculture: applications and trends. J Proteomics.

[14] Addis MF, Cappuccinelli R, Tedde V, Pagnozzi D, Porcu MC, Bonaglini E, Roggio T, Uzzau S (2010). Proteomic analysis of muscle tissue from gilthead sea bream (Sparus aurata, L.) farmed in offshore floating cages. Aquaculture.

[15] Martin SAM, Cash P, Blaney S, Houlihan DF (2001). Proteome analysis of rainbow trout (*Oncorhynchus mykiss*) liver proteins during short term starvation. Fish Physiol Biochem.

[16] Vilhelmsson OT, Martin SAM, Poli BM, Houlihan DF (2003). Proteomics: Methodology and application in fish processing.

[17] Douxfils J, Mathieu C, Mandiki SNM, Milla S, Henrotte E, Wang N, Vandecan M, Dieu M, Dauchot N, Pigneur L-M, Li X, Rougeot C, Mélard C, Silvestre F, Van Doninck K, Raes M, Kestemont P (2011). Physiological and proteomic evidences that domestication process differentially modulates the immune status of juvenile Eurasian perch (*Perca fluviatilis*) under chronic confinement stress. Fish Shellfish Immunol.

[18] Alves RN, Cordeiro O, Silva TS, Richard N, de Vareilles M, Marino G, Di Marco P, Rodrigues PM, Conceição LEC (2010). Metabolic molecular indicators of chronic stress in gilthead seabream (*Sparus aurata*) using comparative proteomics. Aquaculture.

[19] Braceland M, Bickerdike R, Tinsley J, Cockerill D, McLoughlin MF, Graham D a, Burchmore RJ, Weir W, Wallace C, Eckersall PD (2013). The serum proteome of Atlantic salmon, *Salmo salar*, during pancreas disease (PD) following infection with salmonid alphavirus subtype 3 (SAV3). J Proteomics.

[20] Addis MF, Cappuccinelli R, Tedde V, Pagnozzi D, Viale I, Meloni M, Salati F, Roggio T, Uzzau S (2010). Influence of Moraxella sp. colonization on the kidney proteome of farmed gilthead sea breams (Sparus aurata, L.). Proc Natl Acad Sci U S A.

[21] Brunt J, Hansen R, Jamieson DJ, Austin B (2008). Proteomic analysis of rainbow trout (*Oncorhynchus mykiss*, Walbaum) serum after administration of probiotics in diets. Vet Immunol Immunopathol.

[22] Ibarz A, Martín-Pérez M, Blasco J, Bellido D, de Oliveira E, Fernández-Borràs J (2010). Gilthead sea bream liver proteome altered at low temperatures by oxidative stress. Proteomics.

[23] Varó I, Navarro JC, Rigos G, Del Ramo J, Calduch-Giner JA, Hernández A, Pertusa J, Torreblanca A (2013). Proteomic evaluation of potentiated sulfa treatment on gilthead sea bream (Sparus aurata L.) liver. Aquaculture.

[24] Isani G, Andreani G, Carpenè E, Di Molfetta S, Eletto D, Spisni E (2011). Effects of waterborne Cu exposure in gilthead sea bream (*Sparus aurata*): a proteomic approach. Fish Shellfish Immunol.

[25] Biosa G, Addis MF, Tanca A, Pisanu S, Roggio T, Uzzau S, Pagnozzi D (2011). Comparison of blood serum peptide enrichment methods by Tricine SDS-PAGE and mass spectrometry. J Proteomics.

[26] Terova G, Pisanu S, Roggio T, Preziosa E, Saroglia M, Addis MF (2014). Proteomic profiling of sea bass muscle by two-dimensional gel electrophoresis and tandem mass spectrometry. Fish Physiol Biochem.

[27] Addis MF, Pisanu S, Ghisaura S, Pagnozzi D, Marogna G, Tanca A, Biosa G, Cacciotto C, Alberti A, Pittau M, Roggio T, Uzzau S (2011). Proteomics and pathway analyses of the milk fat globule in sheep naturally infected by *Mycoplasma agalactiae* provide indications of the in vivo response of the mammary epithelium to bacterial infection. Infect Immun.

[28] Choudhury M, Yamada S (2009). Homologue of mammalian apolipoprotein A-II in non-mammalian vertebrates. Acta Biochim Biophys Sin.

[29] Li CJ, Gan F, Chen XH, Liu ZG, Li LX, Wei QW, Tang YK (2011). Molecular and expression analysis of apolipoprotein E gene in the Chinese sturgeon, *Acipenser sinensis*. Comp Biochem Physiol B Biochem Mol Biol.

[30] Bernlohr DA, Simpson MA, Hertzel AV, Banaszak LJ (1997). Intracellular lipid-binding proteins and their genes. Annu Rev Nutr.

[31] Hertzel AV, Bernlohr DA (2000). The mammalian fatty acid-binding protein multigene family: molecular and genetic insights into function. Trends Endocrinol Metab.

[32] Moyano FJ, Cardenete G, De la Higuera M (1991). Nutritive and metabolic utilization of proteins with high glutamic acid content by the rainbow trout (*Oncorhynchus mykiss*). Comp Biochem Physiol Part A Physiol.

[33] Ji C, Kaplowitz N (2003). Betaine decreases hyperhomocysteinemia, endoplasmic reticulum stress, and liver injury in alcohol-fed mice. Gastroenterology.

[34] Xiao T, Shoeb M, Siddiqui MS, Zhang M, Ramana KV, Srivastava SK, Vasiliou V, Ansari NH (2009). Molecular cloning and oxidative modification of human lens ALDH1A1: implication in impaired detoxification of lipid aldehydes. J Toxicol Environ Health A.

[35] Kikuchi K, Yamashita M, Watabe S, Aida K (1995). The warm temperature acclimation-related 65-kDa protein, Wap65, in goldfish and its gene expression. J Biol Chem.

[36] Sha Z, Xu P, Takano T, Liu H, Terhune J, Liu Z (2008). The warm temperature acclimation protein Wap65 as an immune response gene: its duplicates are differentially regulated by temperature and bacterial infections. Mol Immunol.

[37] Shi YH, Chen J, Li CH, Li MY (2010). Molecular cloning of liver Wap65 cDNA in ayu (*Plecoglossus altivelis*) and mRNA expression changes following *Listonella anguillarum* infection. Mol Biol Rep.

[38] Clark MS, Burns G (2009). Characterisation of the warm acclimated protein gene (wap65) in the Antarctic plunderfish (*Harpagifer antarcticus*). Mitochondrial DNA.

[39] Wicher KB, Fries E (2010). Evolutionary aspects of hemoglobin scavengers. Antioxid Redox Signal.

[40] Bayne CJ, Gerwick L (2001). The acute phase response and innate immunity of fish. Dev Comp Immunol.

[41] Janciauskiene S, Larsson S, Larsson P, Virtala R, Jansson L, Stevens T (2004). Inhibition of lipopolysaccharide-mediated human monocyte activation, in vitro, by α1-antitrypsin. Biochem Biophys Res Commun.

[42] Bergin DA, Reeves EP, Meleady P, Henry M, McElvaney OJ, Carroll TP, Condron C, Chotirmall SH, Clynes M, O’Neill SJ, McElvaney NG (2010). α-1 Antitrypsin regulates human neutrophil chemotaxis induced by soluble immune complexes and IL-8. J Clin Invest.

[43] Magnadottir B, Audunsdottir SS, Bragason BT, Gisladottir B, Jonsson ZO, Gudmundsdottir S (2011). The acute phase response of Atlantic cod (*Gadus morhua*): Humoral and cellular response. Fish Shellfish Immunol.

[44] Talbot AT, Pottinger TG, Smith TJ, Cairns MT (2009). Acute phase gene expression in rainbow trout (*Oncorhynchus mykiss*) after exposure to a confinement stressor: A comparison of pooled and individual data. Fish Shellfish Immunol.

[45] Lü JN, Chen J, Lu XJ, Shi YH (2012). Identification of α1-antitrypsin as a positive acute phase protein in ayu (*Plecoglossus altivelis*) associated with *Listonella anguillarum* infection. Fish Shellfish Immunol.

[46] Ellis AE (2001). Innate host defense mechanisms of fish against viruses and bacteria. Dev Comp Immunol.

[47] Stafford JL, Belosevic M (2003). Transferrin and the innate immune response of fish: identification of a novel mechanism of macrophage activation. Dev Comp Immunol.

[48] Stafford JL, Neumann NF, Belosevic M (2001). Products of proteolytic cleavage of transferrin induce nitric oxide response of goldfish macrophages. Dev Comp Immunol.

[49] Salerno G, Parisi MG, Parrinello D, Benenati G, Vizzini A, Vazzana M, Vasta GR, Cammarata M (2009). F-type lectin from the sea bass (*Dicentrarchus labrax*): purification, cDNA cloning, tissue expression and localization, and opsonic activity. Fish Shellfish Immunol.

[50] Cammarata M, Benenati G, Odom EW, Salerno G, Vizzini A, Vasta GR, Parrinello N (2007). Isolation and characterization of a fish F-type lectin from gilt head bream (*Sparus aurata*) serum. Biochim Biophys Acta.

[51] Folch J, Lees M, Sloane Stanley GH (1957). A simple method for the isolation and purification of total lipides from animal tissues. J Biol Chem.

[52] Wiśniewski JR, Zielinska DF, Mann M (2011). Comparison of ultrafiltration units for proteomic and N-glycoproteomic analysis by the filter-aided sample preparation method. Anal Biochem.

[53] Tanca A, Palomba A, Deligios M, Cubeddu T, Fraumene C, Biosa G, Pagnozzi D, Addis MF, Uzzau S (2013). Evaluating the Impact of Different Sequence Databases on Metaproteome Analysis: Insights from a Lab-Assembled Microbial Mixture. PLoS One.

[54] Tanca A, Addis MF, Pagnozzi D, Cossu-Rocca P, Tonelli R, Falchi G, Eccher A, Roggio T, Fanciulli G, Uzzau S (2011). Proteomic analysis of formalin-fixed, paraffin-embedded lung neuroendocrine tumor samples from hospital archives. J Proteomics.

[55] Zhang Y, Wen Z, Washburn MP, Florens L (2010). Refinements to label free proteome quantitation: how to deal with peptides shared by multiple proteins. Anal Chem.

[56] Melis R, Anedda R (2014). Biometric and metabolic profiles associated to different rearing conditions in offshore farmed gilthead sea bream (Sparus aurata L.). Electrophoresis.

[57] Melis R, Cappuccinelli R, Roggio T, Anedda R: **Addressing marketplace gilthead sea bream (*****Sparus aurata*****L.) differentiation by 1H NMR-based lipid fingerprinting.** *Food Res Int*. In press, doi:10.1016/j.foodres.2014.05.041.

[58] Terova G, Addis MF, Preziosa E, Pisanu S, Pagnozzi D, Biosa G, Gornati R, Bernardini G, Roggio T, Saroglia M (2011). Effects of postmortem storage temperature on sea bass (*Dicentrarchus labrax*) muscle protein degradation: Analysis by 2-D DIGE and MS. Proteomics.

[59] Westermeier R, Naven T, Höpker H-R (2008). Proteomics in Practice: A Guide to Successful Experimental Design.

[60] Addis MF, Tanca A, Pagnozzi D, Rocca S, Uzzau S (2009). 2-D PAGE and MS analysis of proteins from formalin-fixed, paraffin-embedded tissues. Proteomics.

