# Supplementary material for: Impact of three commercial feed formulations on farmed gilthead sea bream (Sparus aurata, L.) metabolism as inferred from liver and blood serum proteomics
Source: Proteome Sci. 2014 Sep 24;12:44. doi: 10.1186/s12953-014-0044-3 (PMC4200174; doi:10.1186/s12953-014-0044-3)
Supplement: Additional file 2: — Detailed protein identifications of liver. [file 12953_2014_44_MOESM2_ESM.pdf]

Additional file 2. Protein expression trends observed for T12A, T12B, and T12C vs T0. Spots are numbered according to Figure 2.

Spot	T12A vs T0	T12B vs T0	T12C vs T0	P value	Protein Name	Species	Acc. No.[a]	MW[b]	pI[c]	Score[d]	C (%)[e]	UP[f]	P[g]
1		1.7		0.0038	Heat shock protein	*E. akaara*	F4ZL29	61.2	5.6	747.7	48.3	2	30
7		1.52		0.0039	UTP-glucose-1-phosphate uridylyltransferase	*O. niloticus*	I3KGA9	56.5	8.5	127.9	14.1	2	6
8		1.83		0.0016	Alpha amylase	*P. pagrus*	Q5XPY5	35.2	7.9	148.3	20.3	2	6
9		1.77		0.0043	Alpha amylase	*P. pagrus*	Q5XPY5	35.2	7.9	280.7	29.4	2	9
					UTP-glucose-1-phosphate uridylyltransferase	*O. niloticus*	I3KGA9	56.5	8.5	156.9	26.8	5	13
10		1.74		0.0026	UTP-glucose-1-phosphate uridylyltransferase	*P. flavescens*	F1C734	47.1	8.4	271.1	47.3	2	24
12		1.55		0.002	Elongation factor 1-alpha	*S. aurata*	Q9PUG6	50.6	9.2	310.1	52.9	8	24
13			1.65	0.047	Betaine homocysteine methyltransferase	*S. aurata*	Q1KMD2	44.3	7.6	622.2	60.6	15	18
14			1.61	0.047	Betaine homocysteine methyltransferase	*S. aurata*	Q1KMD2	44.3	7.6	451.7	70.3	18	23
15	1.52	-4.58	2.01	0.0054	Adenosylhomocysteinase	*S. salar*	B5DGE0	47.9	6.9	137.6	17.1	2	7
16	-2.02			0.0058	Fumarylacetoacetate hydrolase	*D. rerio*	Q803S0	38.7	6.7	62.4	13.5	3	4
17	1.53			0.0086	4-hydroxyphenylpyruvate dioxygenase	*A. fimbria*	C3KIF6	45.1	6.8	124.0	24.6	4	7
18		1.63	1.32	0.0024	Isocitrate dehydrogenase	*G. aculeatus*	G3P9W8	47.1	6.9	494.7	39.8	6	17
20	1.77			0.0009	40S ribosomal protein SA	*D. labrax*	K7DWA9	33.7	5.1	489.7	56.7	17	33
22	2			0.096	Malate dehydrogenase	*G. aculeatus*	G3NH26	38.6	7	126.8	16.6	6	6
23		-3.18		0.003	Fructose-bisphosphate aldolase	*E. coioides*	G8G904	39.3	8.2	117.8	15.1	3	6
24	1.79	-1.91		0.003	Fructose-bisphosphate aldolase	*E. coioides*	G8G904	39.3	8.2	509.0	22.0	5	13
27		-2.08		0.0013	Fructose-bisphosphate aldolase	*E. coioides*	G8G904	39.3	8.2	374.3	24.2	4	14
28		-1.83		0.005	Fructose-bisphosphate aldolase	*E. coioides*	G8G904	39.3	8.2	78.5	14.0	2	4
29		-2.08		0.0025	Malate dehydrogenase	*T. nigroviridis*	H3CSR4	36.33	6.7	142.1	8.8	2	2
					Betaine homocysteine methyltransferase	*S. aurata*	Q1KMD2	44.26	7.6	110.5	12.7	3	2
30	1.64			0.0024	Fructose-bisphosphate aldolase	*E. coioides*	G8G904	39.3	8.2	469.4	26.4	4	14
31	-1.59	-1.73		0.0039	Guanine nucleotide-binding protein subunit beta-2-like 1	*A. fimbria*	C3KHC5	35.0	7.2	182.1	41.0	3	11
33		-2.06	-1.62	0.009	Prohibitin	*G. aculeatus*	G3P3A6	29.8	5.4	91.9	15.1	4	4
					High choriolytic enzyme 1	*A. fimbria*	C3KIX2	30.6	5.4	84.7	11.9	3	4
34	-1.57			0.0007	3-hydroxyanthranilate 34-dioxygenase	*P. flavescens*	F1C761	24.8	6.4	75.0	15.0	4	5
36	1.69			0.0096	Betaine homocysteine methyltransferase	*S. aurata*	Q1KMD2	44.3	7.6	97.3	26.2	5	6
37			-1.8	0.047	Apolipoprotein A-IV	*G. aculeatus*	G3NCX3	25.49	5.7	51.8	5.7	2	2
38	1.89			0.0007	Apolipoprotein A-1	*P. major*	Q6Y255	22.4	6.0	90.0	14.7	2	3
39	1.66			0.0088	Apolipoprotein A-1	*P. major*	Q6Y255	22.4	6.0	105.3	14.7	4	4
40	1.75			0.0045	Betaine homocysteine methyltransferase	*S. aurata*	Q1KMD2	44.3	7.6	248.6	49.4	16	17
41		-2.1	1.41	0.0044	Betaine homocysteine methyltransferase	*S. aurata*	Q1KMD2	44.3	7.6	154.2	25.9	14	14
42	-2.34	1.55		0.0053	Peptidyl-prolyl cis-trans isomerase	*P. maxima*	A0F006	16.1	8.2	105.3	23.3	3	4
43	1.9	-2.17		0.0011	Fatty acid binding protein-like protein	*S. aurata*	Q4QY75	14.1	8.3	49	37.3	2	2
44	1.7			0.0045	Alpha-2 globin	*S. aurata*	Q19LH2	15.8	8.7	551.3	62.2	12	12
45	-1.35	-4.35	-1.95	0.0043	Alpha-1 globin	*S. aurata*	Q19LH3	16.0	9.4	204.1	31.9	7	7
46	2.02	1.59		0.0009	14KDa Apolipoprotein	*S. aurata*	Q4QY86	15.9	5.3	273.7	51.8	7	7
47		1.77		0.0033	14KDa apolipoprotein	*S. aurata*	Q4QY86	15.86	5.3	60.5	35.7	4	4
					Nucleoside diphosphate kinase	*O. niloticus*	I3K7K3	13.26	9.3	38.9	21.9	2	2
49	-2.2	-7.18	-1.87	0.0013	Alpha-2 globin	*S. aurata*	Q19LH2	15.83	8.7	101.5	64.3	7	7

a Accession number.
b Calculated molecular weight of full-length protein in kDa.
c Theoretical isoelectric point.
d Score: probability score in Mascot program (the probability that the observed match between the experimental data and mass values calculated from a candidate peptide sequence is a random event.
ePercent coverage: the minimum coverage of the matched peptide in relation to the full-length sequence.
[f]Unique peptides
gNumber of peptides
