# Supplementary material for: Impact of three commercial feed formulations on farmed gilthead sea bream (Sparus aurata, L.) metabolism as inferred from liver and blood serum proteomics
Source: Proteome Sci. 2014 Sep 24;12:44. doi: 10.1186/s12953-014-0044-3 (PMC4200174; doi:10.1186/s12953-014-0044-3)
Supplement: Additional file 3: — Protein networks generated by IPA software. [file 12953_2014_44_MOESM3_ESM.pdf]

Additional file 3. Top scoring networks according to IPA analysis of liver proteins. Panels A, B, and C report the top scoring networks obtained for Feed A, B, and C, respectively. A: cell-to-cell signaling and interaction, inflammatory response, lipid metabolism; B: left, cell-to-cell signaling and interaction, cellular function and maintenance, inflammatory response; right, cell death and survival, cellular compromise, cell cycle. C: lipid metabolism, molecular transport, small molecule biochemistry. Red, upregulated proteins; green, downregulated proteins; white, proteins indicated by IPA as significantly associated with the reported network but not identified in our study. Lines connecting the molecules indicate molecular relationships: continuous line, direct relationship; dashed line, indirect relationship. Color intensity represents the extent of differential protein abundance. Expression values are overlayed to the network. Please refer to Table 5 for protein abbreviations.

Legend to networks:

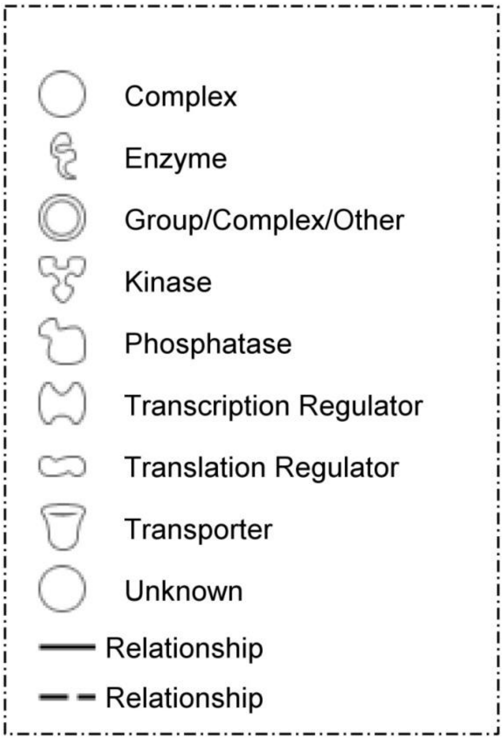

Network 1, T12A vs T0:

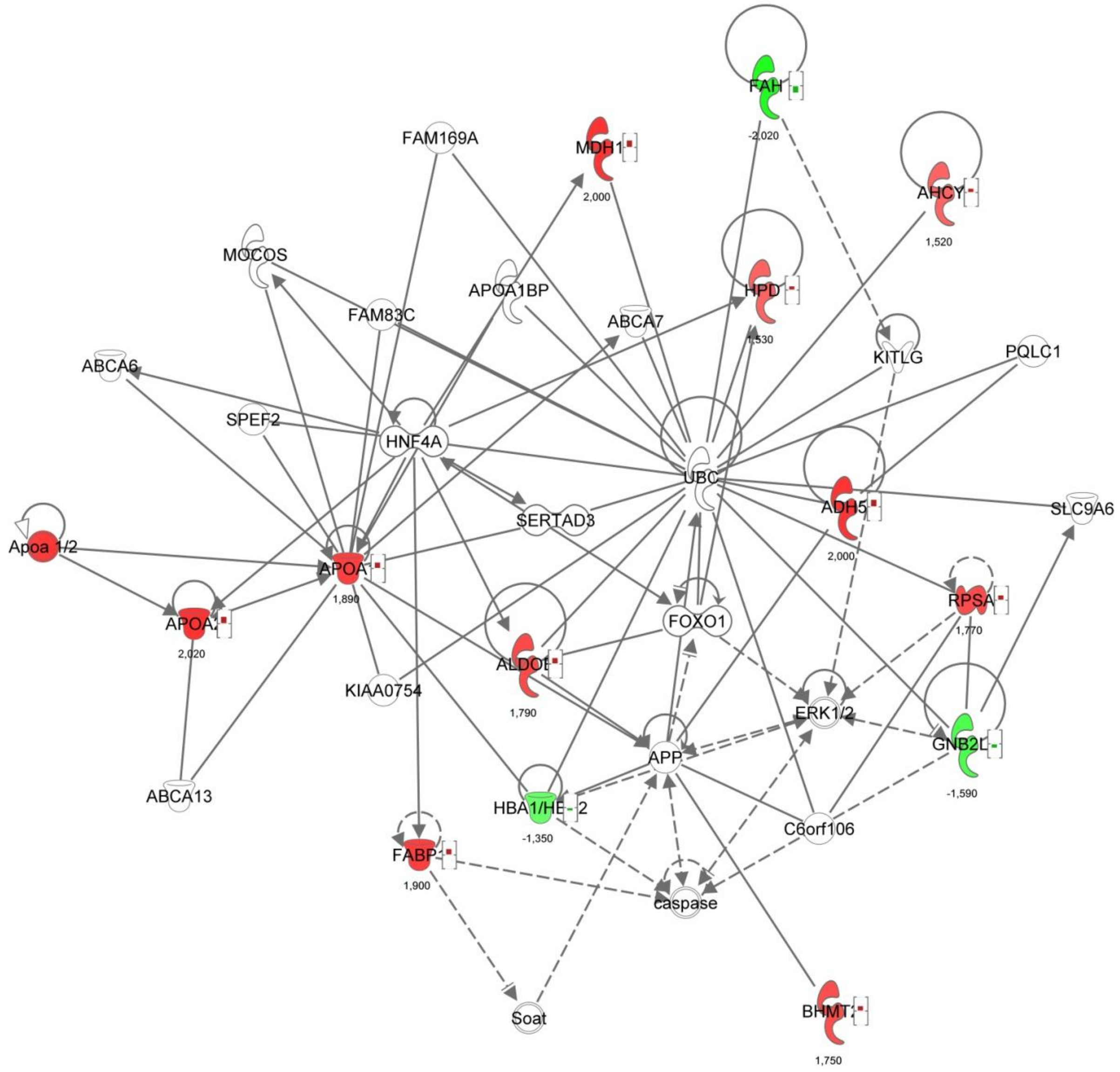

© 2000-2013 Ingenuity Systems, Inc. All rights reserved.

Network 1, T12B vs T0:

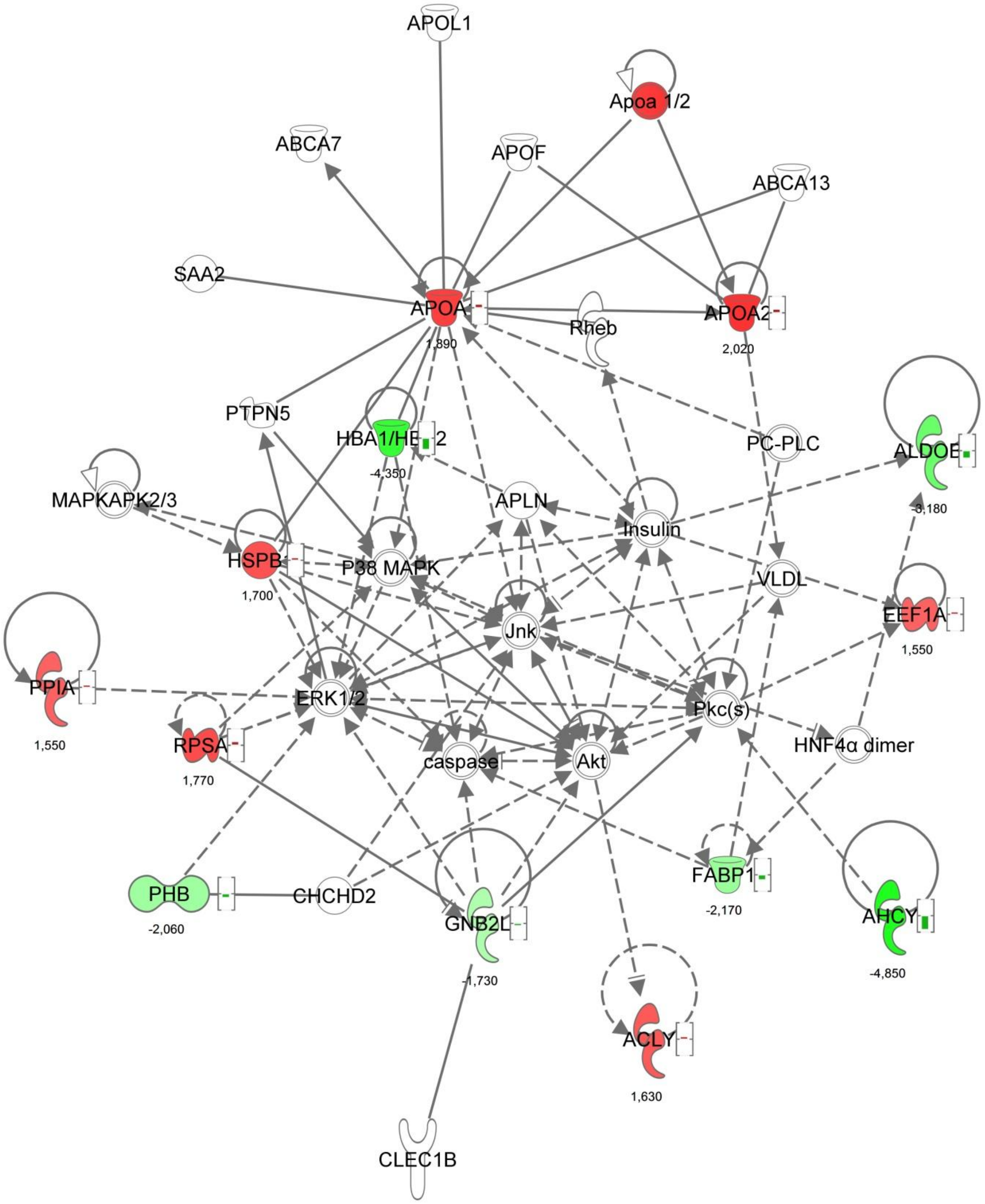

© 2000-2013 Ingenuity Systems, Inc. All rights reserved.

Network 2, T12B vs T0:

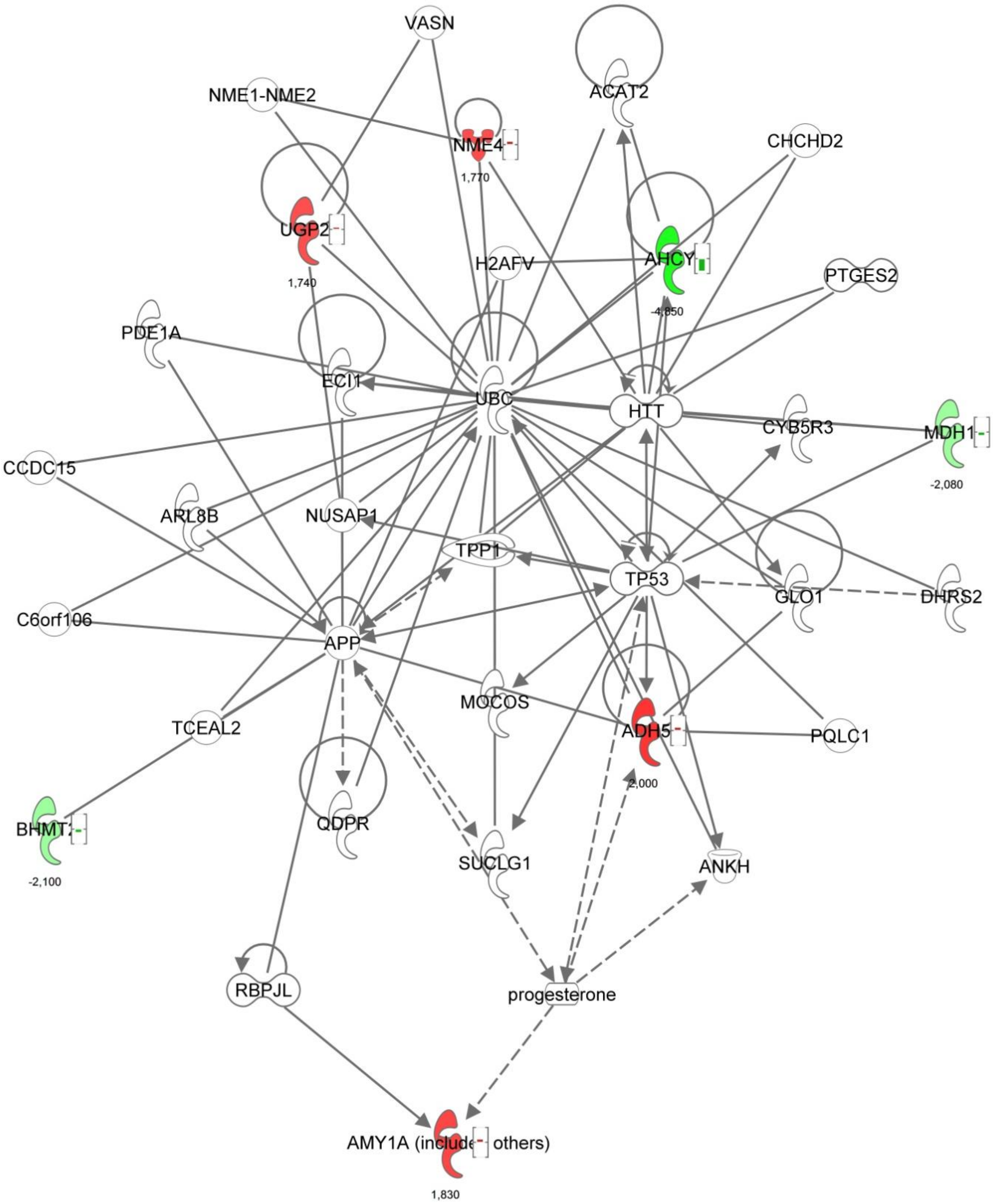

© 2000-2013 Ingenuity Systems, Inc. All rights reserved.

Network 1, T12C vs T0:

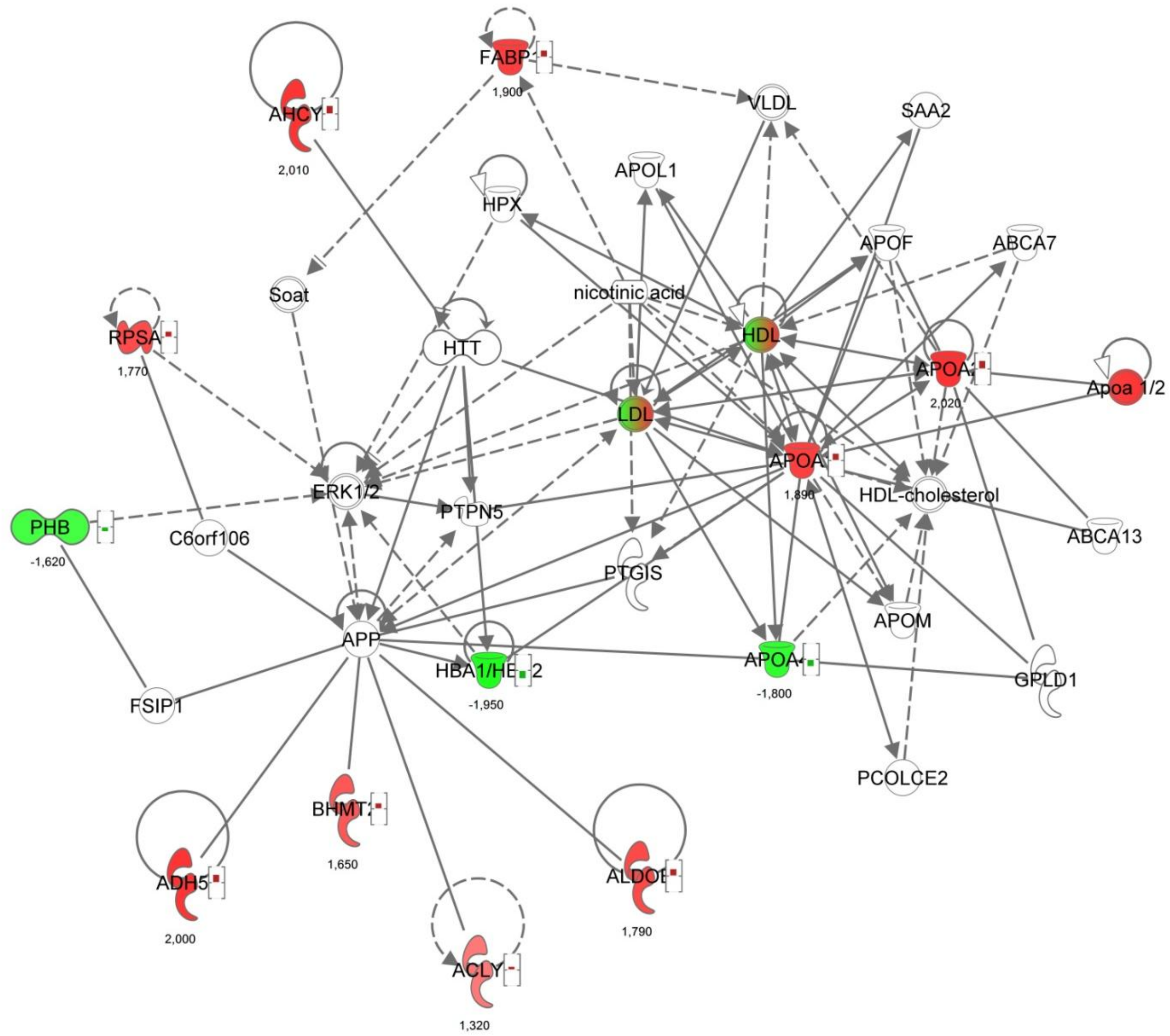

© 2000-2013 Ingenuity Systems, Inc. All rights reserved.
