# Supplementary material for: Impact of three commercial feed formulations on farmed gilthead sea bream (Sparus aurata, L.) metabolism as inferred from liver and blood serum proteomics
Source: Proteome Sci. 2014 Sep 24;12:44. doi: 10.1186/s12953-014-0044-3 (PMC4200174; doi:10.1186/s12953-014-0044-3)
Supplement: Additional file 4: — Detailed protein identifications of serum for the comparison of T12A, T12B and T12C vs T0. [file 12953_2014_44_MOESM4_ESM.pdf]

Additional file 4. Detailed protein identifications of serum for the comparison of T12A, T12B and T12C vs T0. Spots with average ratios +/- 1.5 are indicated in bold. Spots are numbered according to Figure 5. Please note that spots that did not provide a valid protein identification by mass spectrometry are not reported.

**Spot**	**T12A vs T0**	**T12B vs T0**	**T12C vs T0**	**P value**	**Protein Name**	**Species**	**Acc. No.[a]**	**MW[b]**	**pI[c]**	**Score[d]**	**%C[e]**	**UP[f]**	**P[g]**
1	**2.3**			0.00016	Alpha 1 antitrypsin	*S. aurata*	Q4QY84	29.6	5.1	244.6	44.1	9	11
2	**1.7**			1.1E-06	Alpha 1 antitrypsin	*S. aurata*	Q4QY84	29.6	5.1	240.5	29.7	6	6
3		**1.5**		0.000011	Transferrin (fragments)	*S. aurata*	F2YLA1	74.2	6.3	225.2	11.6	4	4
4		**1.9**		0.00016	Fibrinogen beta chain	*L. crocea*	I3K9D6	57.8	7.7	396.1	16.4	2	10
5	**1.8**	**2.0**	1.3	0.000055	Fibrinogen beta chain	*G. aculeatus*	G3Q4A3	55.1	7.0	473.1	18.9	4	11
6	1.4		**1.6**	0.0081	Fibrinogen beta chain	*P. olivaceus*	A5JV29	55.7	6.9	95.4	6.7	2	4
7		**1.5**		0.0016	14 kDa apolipoprotein	*S. aurata*	Q4QY86	15.9	5.3	40.0	35.7	3	3
8	**1.5**	1.3	1.3	0.000088	14 kDa apolipoprotein	*S. aurata*	Q4QY86	15.9	5.3	63.6	11.9	2	2
9	**1.5**			0.00021	14 kDa apolipoprotein	*S. aurata*	Q4QY86	15.9	5.3	78.67	11.9	2	2

[a]Accession number.
[b]Calculated molecular weight of full-length protein in kDa.
[c]Theoretical isoelectric point.
[d]Score: probability score in Mascot program (the probability that the observed match between the experimental data and mass values calculated from a candidate peptide sequence is a random event.
[e]Percent coverage: the minimum coverage of the matched peptide in relation to the full-length sequence.
[f]Unique peptides
[g]Number of peptides
