# Supplementary material for: Impact of three commercial feed formulations on farmed gilthead sea bream (Sparus aurata, L.) metabolism as inferred from liver and blood serum proteomics
Source: Proteome Sci. 2014 Sep 24;12:44. doi: 10.1186/s12953-014-0044-3 (PMC4200174; doi:10.1186/s12953-014-0044-3)
Supplement: Additional file 5: — Detailed protein identifications of serum for the comparison of T12A vs T12B. [file 12953_2014_44_MOESM5_ESM.pdf]

Additional file 5. Protein spots showing statistically significant differences in expression between T12A and T12B. Spots are numbered according to Figure 7.

Spot	Av. Ratio T12A/T12B	P value	Protein Name	Species	Acc. No.[a]	MW[b]	pI[c]	Score[d]	%C[e]	UP[f]	P[g]
1	1.59	0.000043	Complement component c3	*S. aurata*	F8R6K2	185.2	7.9	39.9	2.4	4	4
2	3.14	0.00016	Alpha 1 antitrypsin	*S. aurata*	Q4QY84	29.6	5.1	244.6	44.11	9	11
			Warm temperature acclimation-related 65 kDa protein	*S. aurata*	C0L788	49.1	5.8	149.0	31.5	6	12
3	1.51	1.1E-06	Warm temperature acclimation-related 65 kDa protein	*S. aurata*	C0L788	49.1	5.8	801.6	48.2	17	18
4	2.96	0.0013	Warm temperature acclimation-related 65 kDa protein	*S. aurata*	C0L788	49.1	5.8	249.8	36.2	7	16
			Alpha 1 antitrypsin	*S. aurata*	Q4QY84	29.6	5.1	222.8	38.8	5	7
5	1.5	1.1E-06	Warm temperature acclimation-related 65 kDa protein	*S. aurata*	C0L788	49.1	5.8	561.9	28.94	8	8
			Alpha 1 antitrypsin	*S. aurata*	Q4QY84	29.6	5.1	121.02	13.69	2	2
6	1.87	0.000016	Alpha 1 antitrypsin	*S. aurata*	Q4QY84	29.6	5.1	820.7	61.6	12	15
7	3.86	1.6E-07	Alpha 1 antitrypsin	*S. aurata*	Q4QY84	29.6	5.1	775.1	59.3	12	14
8	-1.86	0.000031	Transferrin (fragments)	*S. aurata*	F2YLA1	74.2	6.3	177.3	8.8	4	4
9	-2.25	0.000011	Transferrin (fragments)	*S. aurata*	F2YLA1	74.2	6.3	225.2	11.6	4	4
10	-2.72	0.000055	Fibrinogen beta chain	*G. aculeatus*	G3Q4A3	55.1	7.0	473.1	18.9	4	11
11	1.93	7.4E-06	Apolipoprotein A-1	*P. major*	Q6Y255	22.4	6.0	54.8	14.7	3	3
12	4.73	0.00029	Apolipoprotein A-1	*P. major*	Q6Y255	29.6	5.3	1215.5	70.4	22	22
13	4.19	4.5E-06	Apolipoprotein A-1	*P. major*	Q6Y255	22.4	6.0	77.7	9.6	2	2
14	2.54	5.7E-08	Apolipoprotein A-IV4	*G. aculeatus*	G3NGM9	26.5	4.8	198.1	12.0	3	4
15	-3.3	0.0029	14 kDa apolipoprotein	*S. aurata*	Q4QY86	15.9	5.3	88.4	18.9	3	3
16	1.57	2.1E-06	Transferrin	*S. aurata*	F2YLA1	74.2	6.3	737.8	29.8	16	17
17	1.57	0.000022	Transferrin	*S. aurata*	F2YLA1	74.2	6.3	1464.6	48.3	30	31
18	2.49	3.1E-07	Transferrin	*S. aurata*	F2YLA1	74.2	6.3	1506.3	45.0	27	27
19	-1.77	4,1E-07	F-type lectin 2	*O. fasciatus*	F7J049	34.5	6.3	348.4	13.6	2	5
20	2.14	1,8E-07	F-type lectin 2	*O. fasciatus*	F7J049	34.5	6.3	261.3	13.6	3	5

[a]Accession number.
[b]Calculated molecular weight of full-length protein in kDa.
[c]Theoretical isoelectric point.
[d]Score: probability score in Mascot program (the probability that the observed match between the experimental data and mass values calculated from a candidate peptide sequence is a random event.
[e]Percent coverage: the minimum coverage of the matched peptide in relation to the full-length sequence.
[f]Unique peptides
[g]Number of peptides
